# 3D Bioprinted Engineered Living Microreactors for Continuous Organophosphorus Compound Degradation

**DOI:** 10.1002/smsc.202500226

**Published:** 2025-09-04

**Authors:** Mark R. Shannon, Graham J. Day, Hermes Bloomfield‐Gadêlha, Valeska P. Ting, Adam W. Perriman

**Affiliations:** ^1^ School of Cellular and Molecular Medicine University of Bristol University Walk Bristol BS8 1TD UK; ^2^ Bristol Composites Institute School of Civil, Aerospace and Design Engineering University of Bristol Bristol BS8 1TR UK; ^3^ School of Engineering Mathematics and Technology University of Bristol Tankard's Close Bristol BS8 1TW UK; ^4^ Bristol Robotics Laboratory (BRL) UWE Bristol T‐Block Bristol BS16 1QY UK; ^5^ Research School of Chemistry Australian National University Building 137 Sullivans Creek Rd, Acton Canberra ACT 2601 Australia; ^6^ John Curtin School of Medical Research Australian National University 131 Garran Rd Acton Canberra ACT 2601 Australia

**Keywords:** 3D printing, biomaterials, bioreactors, bioremediation, engineered living materials, environmental biotechnology, pesticides

## Abstract

Engineered living materials (ELMs) are poised to play a pivotal role in addressing critical global environmental challenges through advances in green energy production, biosensing and bioremediation. When coupled with advanced manufacturing techniques, such as 3D bioprinting, new opportunities emerge for the fabrication of high‐resolution self‐supporting ELM structures suitable for hosting microbial populations with bespoke chemical activity. Accordingly, the design and fabrication of a 3D bioprinted microbial ELM flow‐bioreactor comprising genetically engineered *Escherichia coli* are described. The metabolically active ELM bioreactor cyclically detoxifies organophosphorus compounds via inducible expression of the *Agrobacterium radiobacter* phosphotriesterase. Principal component analysis is performed to reduce the dimensionality of the mass transfer kinetic analysis, uncovering spatiotemporal features within the dynamical evolution of the data. This provides valuable insights into the design parameters essential for the development of highly efficient catalytic microbial ELM bioreactors.

## Graphical abstract

3D bioprinted engineered living microreactors offer a novel solution for continuous organophosphorus compound (OPC) degradation. This work details the fabrication and characterization of porous microbial ELM bioreactors containing genetically engineered *Escherichia coli* for cyclic OPC detoxification. Mass transfer kinetics are analyzed, revealing crucial design parameters for optimizing catalytic efficiency and promising advancements in environmental biotechnology and bioremediation.

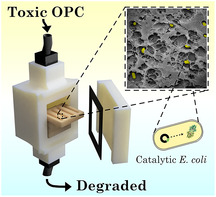

## 1 Introduction

Engineered living materials (ELMs) are a new class of functional materials with significant potential to bridge the sustainable outputs of emerging bioeconomies to practical, real‐world applications.^[^ ^1^, ^2^, ^3^ ^]^ When fabricated using genetically engineered microbial cell populations, ELMs can deliver an impressive range of functionalities.^[^ ^4^ ^]^ These include catalytic bioremediation^[^ ^5^, ^6^ ^]^ and biomolecule production,^[^ ^7^, ^8^, ^9^ ^]^ small molecule^[^ ^10^ ^]^ and light^[^ ^11^ ^]^ sensing, self‐healing through mineral deposition,^[^ ^12^, ^13^ ^]^ mechanical actuation through hygroscopic swelling^[^ ^14^, ^15^ ^]^ or biomass expansion,^[^ ^16^ ^]^ and electrical current generation using microbial metabolism^[^ ^17^ ^]^ or photosynthesis.^[^ ^18^ ^]^ Recently, significant efforts have gone into fabricating ELMs with well‐defined 3D geometries, with a strong emphasis on *Saccharomyces* *cerevisiae* for bioethanol generation.^[^ ^9^ ^]^ Here, the benefits of cell densification, surface area optimization, and strain segregation have been highlighted, along with facile separation of catalytic components from products. Catalytically active yeast cells have also been used to improve the mechanical properties of an ELM for ethanol production, enabling high‐resolution 3D printing.^[^ ^19^ ^]^ This work highlights the benefits of 3D printing‐enabled strain segregation for consortium biosynthesis, suggesting that unwanted competition between producers can be avoided without laborious optimization.

Bioremediation is a rapidly emerging challenge space within the field of microbial ELMs, where systems have been tested under quiescent and shaken‐batch conditions.^[^ ^5^, ^6^, ^20^ ^]^ Indeed, we recently described the development of 3D printable double network hydrogel ELMs for OPC degradation with temperature‐responsive rate modulation.^[^ ^21^ ^]^ Despite these examples, to our knowledge, no 3D printable catalytically active microbial ELM continuous flow reactors have been described. The development of robust, responsive, flow‐compatible ELMs could provide a step change to the bioremediation space, as it takes advantage of high productivity, enhanced product quality, reduced plant footprint and in‐field manufacturing, and process simplification through continuous operation.^[^ ^22^ ^]^

The removal of toxic organophosphorus compound (OPC) pollution is one contemporary environmental challenge of global concern where flow‐compatible, catalytic microbial ELMs could provide an efficient solution to environmental contamination^[^ ^23^ ^]^ or indeed as a potential prophylactic or treatment postexposure. OPCs are acetylcholinesterase inhibitors ranging in toxicity from insecticides such as parathion to the chemical warfare agents (CWAs) sarin and VX.^[^ ^24^ ^]^ The majority of OPC poisoning occurs in rural or regional areas, with 740 000 pesticide poisonings estimated to occur per year worldwide.^[^ ^25^ ^]^ Current methods for decontamination are either inefficient, requiring further downstream processing of waste, or cause significant damage to affected surfaces and are therefore unusable for patient treatment.^[^ ^26^, ^27^ ^]^ An inexpensive, in‐field, readily fabricable green technology for effective OPC removal would therefore be of great interest.

Phosphotriesterases (PTEs) are a series of enzymes capable of catalyzing the hydrolysis of OPCs, which have been genetically engineered to broaden their substrate specificity and enhance their OPC‐degrading capability, expression rates, and thermostability.^[^ ^28^ ^]^ PTEs have been combined with polymers via immobilization^[^ ^29^ ^]^ and direct incorporation^[^ ^30^ ^]^ to develop hybrid materials that can detoxify OPCs. When compared with these hybrid PTE materials, however, a 3D printable ELM microreactor capable of expressing PTE presents significant advantages, which include continual catalyst production, along with the high levels of enzyme efficiency arising from the in vivo environment.^[^ ^31^ ^]^

Whole‐cell systems for OPC degradation have been fabricated using *Escherichia coli* expressing organophosphorus hydrolase. Cells have been immobilized on porous glass beads,^[^ ^32^ ^]^ trapped within alginate suspensions,^[^ ^33^ ^]^ beads,^[^ ^34^ ^]^ within a poly(vinyl alcohol) cryogel,^[^ ^35^ ^]^ and immobilized on cellulosic materials.^[^ ^29^ ^]^ As an alternative, 3D bioprinting represents a significant advance for the fabrication of whole‐cell‐based biocatalytic devices,^[^ ^20^ ^]^ where the desirable properties of a bioink can be optimized for efficient mass transfer rates, biocompatible immobilization of active cell populations, and programmable macroscale geometries.

**Figure 1:**
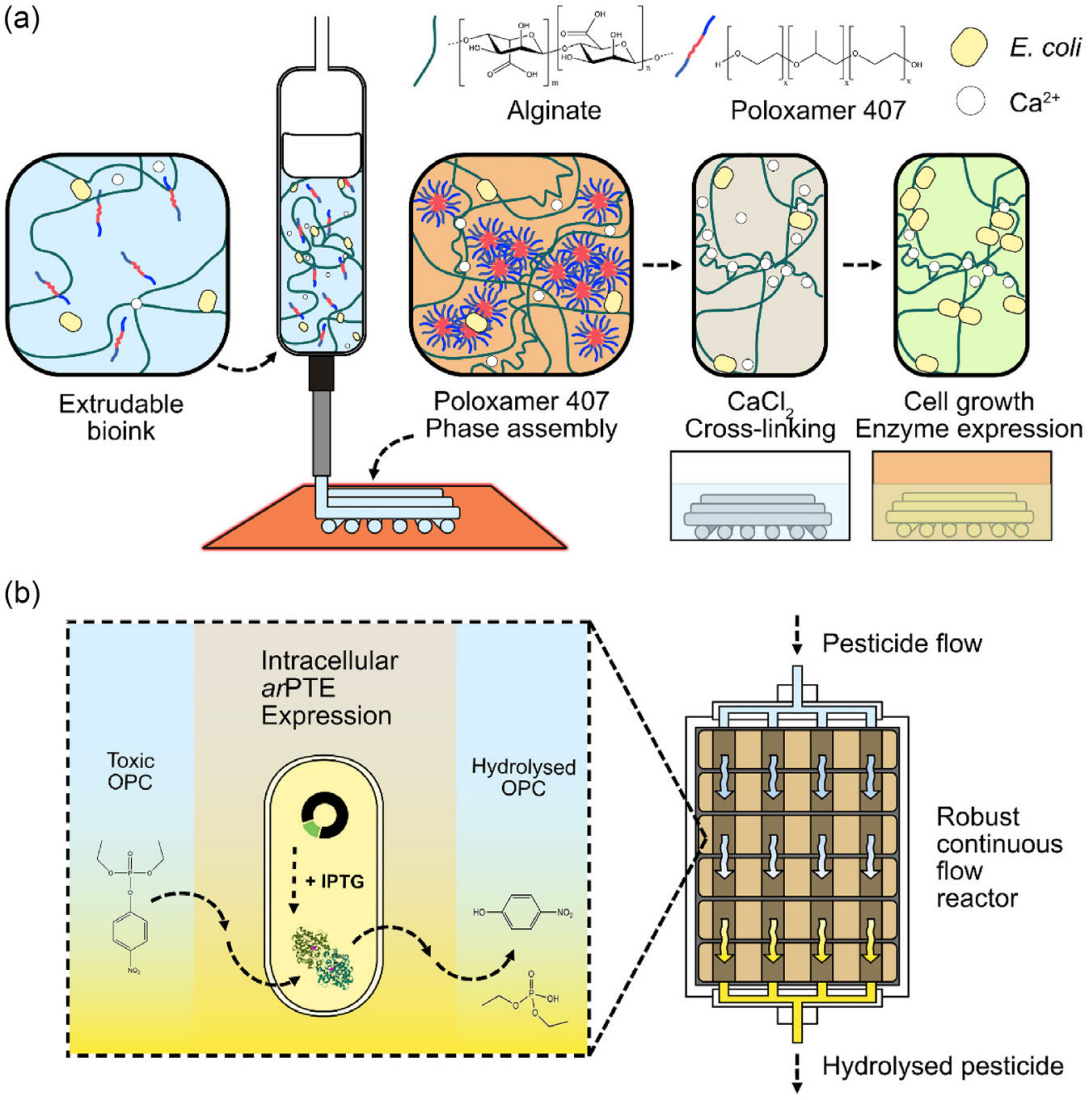
A 3D‐bioprinted, templated‐porous‐alginate, continuous‐flow bioreactor for OPC hydrolysis. a) Schematic of the bioink formulation and the printing process: detailing preprint composition (6 wt% alginate, 13 wt% poloxamer 407, 15 mM CaCl_2_) with *E. coli* cell loading; poloxamer phase assembly on contact with the heated print‐bed; full calcium ion cross‐linking through submersion; further incubation of cross‐linked constructs leading to an increase in cell density within the ELM. b) Introduction of inducible *ar*PTE expression capabilities to the encapsulated *E. coli*, enabling creation of an OPC‐hydrolyzing catalytic ELM that can be assembled into a 3D‐printed continuous flow bioreactor.

The work described herein utilizes an alginate/Poloxamer 407 composite bioink developed to maximize nutrient mass transport in living 3D structures.^[^ ^36^ ^]^ Here, the fugitive porogenic property of the Poloxamer 407 surfactant microphase is exploited to generate microporosity in the hydrogel network, thereby improving small molecule substrate and product diffusion. The composite bioink is then used to 3D bioprint a continuous flow ELM bioreactor comprising metabolically active *E. coli*. By incorporating *E. coli* that can be induced to overexpress *Agrobacterium radiobacter* phosphotriesterase (*ar*PTE) and 3D printing flow‐compatible lattice structures, we have fabricated entirely 3D‐printed flow bioreactors capable of continuous hydrolysis of the insecticide analog paraoxon (**Figure** **1**). Furthermore, we have characterized the reaction diffusion process occurring across the solution/ELM interface, using principal component analysis (PCA) of continuous imaging data to reveal putative design parameters for reactor optimization. This is key if the mass transport limit often identified in immobilized whole‐cell reactor systems is to be addressed and presents an opportunity to capitalize on the robust, biocompatible, and 3D printable ELMs in development.

## 2 Results

### 2.1 Extrusion Printing of a Self‐Supporting, Porous ELM

**Figure 2:**
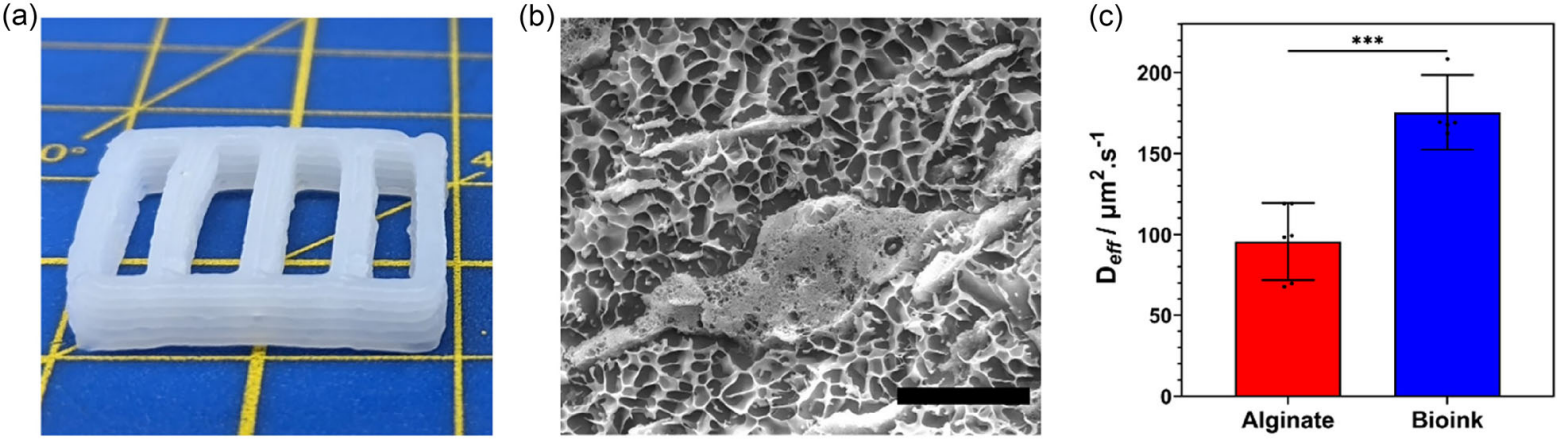
Fabrication of high‐fidelity, reproducible, self‐supporting porous alginate ELM reactor structures. a) Six‐layer flow‐reactor plate immediately after the extrusion printing and cross‐linking process. Grid squares 1 cm. b) Region of templated porous hydrogel with reinforcing dense alginate structure. Scale bar 20 μm. c) Effective diffusion coefficients of 4‐nitrophenolate through cross‐linked 6 wt% alginate and cross‐linked bioink (6 wt% alginate, 13 wt% poloxamer 407, precross‐linked with 15 mM CaCl_2_). Error bars are 95% CI, *** P < 0.001, n = 6.

It was critical to formulate a bioink that could both support the growth of viable bacteria and withstand the shear stress arising from microreactor flow. Moreover, high levels of porosity for maximum substrate and product diffusion were considered within the design criteria. Accordingly, a surfactant‐templated microporous alginate bioink was developed that utilizes a lower critical solution temperature (LCST) phase transition coupled with a Ca^2+^ precross‐linking step. The combination of precross‐linking and a fast transition through the LCST enabled robust, high‐resolution structures to be 3D printed. These included fused discs, 96‐well droplets, multilayer woodpile lattices, single and multifilament rings, and uniform rectangular constructs (Figure S1a–f, Supporting Information). The free‐standing structures were able to be cross‐linked from all but one face by submersion in calcium chloride solution, minimizing swelling during the cross‐linking process. Significantly, robust, high vertical aspect ratio flow‐reactor plates could be readily printed, where the scale was only limited by the syringe volume (**Figure** **2**a). Excellent print reproducibility was confirmed by comparing the masses of cylinders printed using rectilinear and concentric infill patterns in both hydrated and desiccated states (Figure S2, Supporting Information), which was critical for the quantitative comparison of the catalytic ELM structures.

Cryogenic scanning electron microscopy (cryoSEM) of cross‐linked cell‐free printed structures revealed the formation of a microporous network (mean wall separation of 0.86 ± 0.28 μm) reinforced by a second phase of dense alginate material (Figure 2b). Here, the micropores are templated by a Poloxamer 407 microphase, which diffuses from the structure upon cooling during the postprint calcium chloride cross‐linking step.^[^ ^36^ ^]^ The 4‐nitrophenolate colorimetric diffusivity assays showed a near doubling of the effective diffusion rate after surfactant templating (9.6 ± 2.3 × 10^−11^ m^2^ s^−1^ vs 1.76 ± 0.19 ×10^−10^ m^2^ s^−1^ for alginate and the bioink, respectively; Figure 2c, Figure S3, Supporting Information), demonstrating improved mass transfer. For reference, the diffusivity in the bioink was approximately one–fifth of that reported for 4‐nitrophenol through water.^[^ ^37^ ^]^

### 2.2 Encapsulation of Inducible, Metabolically Active *E. coli*

Continuous operation of a catalytic microbial ELM requires maintenance of a metabolically active cell population. To investigate the impact of the bioink on *E. coli* viability, simple disc structures were printed with varied cell loadings (optical density (OD) 0.5, 1.5, and 6.0; Figure S1a, Supporting Information). Syto9/propidium iodide staining of these discs demonstrated the excellent biocompatibility of the templated alginate matrix, with viability calculated by pixel counting (Figure S4, Supporting Information). After printing and cross‐linking, live cells were found to account for 94.1, 80.0, and 96.2% of all cells at loading suspension densities of OD_600_ 0.5, 1.5, and 6.0, respectively. Viability was consistent after 24 h of shaking incubation, increasing up to 96.7, 98.0, and 89.9% for OD_600_ 0.5, 1.5, and 6.0 suspension loadings, respectively (Figure S4a, Supporting Information). Viability was also assessed in ELM structures that had been either induced to produce *ar*PTE by the addition of isopropyl β‐d‐1‐thiogalactopyranoside (IPTG) or repressed with D‐glucose (Figure S4b, Supporting Information). It was found that incubation with D‐glucose supplementation reduced viability (from 87.0 ± 4.4 to 57.0 ± 17.2%), perhaps due to acidification of the media from glucose metabolism, or over‐repression of cyclic adenosine monophosphate production.^[^ ^38^ ^]^ Incubation conditions could likely be optimized to access higher cell densities, for example, using a richer growth medium or through perfusion. For a continuous flow bioreactor, constraint of population growth in this manner is potentially advantageous to prevent both clogging and excessive cell release, as highlighted within the biocoatings field,^[^ ^39^ ^]^ as long as metabolic activity is maintained.

**Figure 3:**
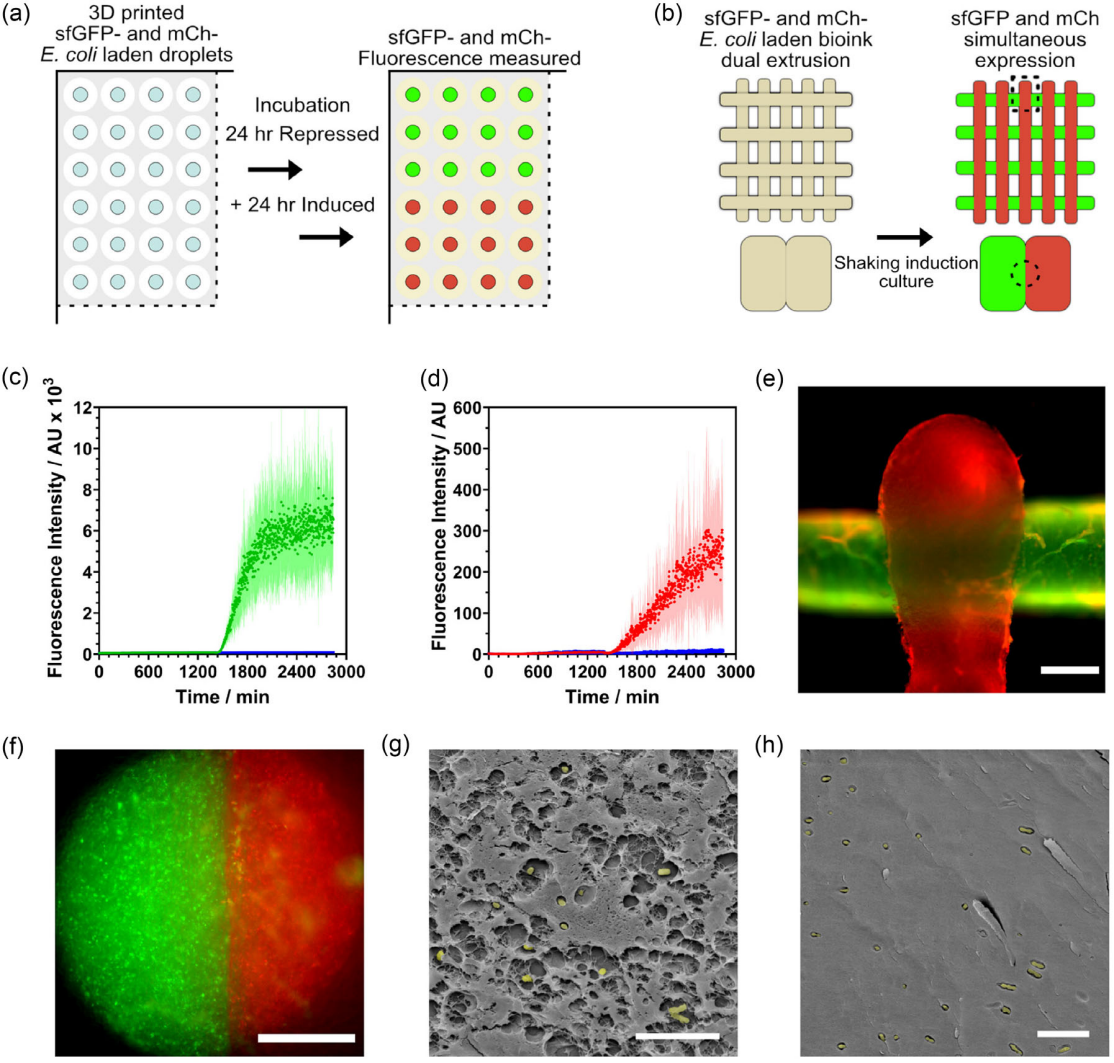
Chemical induction of fluorescent protein production within the 3D bioprinted ELM and SEM analysis of the cell population within the 3D matrix. a) Schematic representation of 96‐well droplet printing used to assess continuous fluorescent protein production under repressing and inducing conditions. b) Schematic of the structures printed using dual extrusion to assess horizontal and vertical transfer of cells from their original filament during culture. c) Fluorescent protein expression of cell laden, cross‐linked 96‐well droplets of bioink in response to chemical induction over 24 h after 24 h of growth with D‐glucose (1w/v%), incubated at 37 °C in storage media A: c) *E. coli*‐sfGFP induced with L‐arabinose (1w/v%) ‐ green, uninduced control ‐ blue. d) *E. coli*‐mCherry induced with IPTG (1 mM) ‐ red, uninduced control ‐ blue. Error bars are ±1 standard deviation, n = 12. e,f) Single‐plane widefield fluorescence microscopy images of dual‐extruded *E. coli*‐sfGFP (green) and *E.coli*‐mCherry (red) lattices; (e) Vertically overlapped filaments. Scale bar 1 mm. (f) Horizontally fused filaments. Scale bar 250 μm. g, h) CryoSEM images of cross‐linked ELM after 24 h of incubation in storage media A. g) Porous region. h) Dense alginate phase. Rod‐like *E. coli*, false colored yellow for clarity, black arrows mark impressions of *E. coli* from the fracture. Scale bars 10 μm.

To further elucidate the state of the bacterial population, strains capable of chemically induced expression of fluorescent proteins were incorporated into the ELM (**Figure** **3**a,b). Significantly, super‐folder green fluorescent protein (sfGFP) or mCherry expression could be induced through the addition of L‐arabinose or IPTG, respectively, to the media surrounding the structures, indicating that small molecules could effectively diffuse through the bioink and that the encapsulated bacteria were metabolically active. Timed induction of protein expression was demonstrated with both sfGFP and mCh‐expressing *E. coli* laden ELM droplets (Figure 3a, Figure S1b, Supporting Information), with tight regulation of expression observed over 24 h with D‐glucose repression, followed by a rapid increase in fluorescence intensity upon addition of inducer (Figure 3c,d).

Widefield fluorescence microscopy of *E. coli‐*sfGFP‐laden 3D‐printed gel constructs further confirmed that small molecules such as L‐arabinose could diffuse through the gel and be taken up by bacterial cells at sufficient levels to sustain a metabolically active population during 24 h of static incubation. An increase in sfGFP signal was observed throughout the hydrogel after one day of growth, followed by L‐arabinose induction of sfGFP expression within the gel, compared to samples imaged immediately after cross‐linking, suggesting that the *E. coli* population grew from a well‐dispersed population observed immediately postprinting (Figure S5a–d, Supporting Information) into distributed clusters of cells (Figure S5e–f, Supporting Information). The growth was also found to align with the printed fiber direction (Figure S6, Supporting Information), perhaps templated along shear‐aligned alginate polymer fibers.^[^ ^36^ ^]^

Filamentation, a characteristic response of *E. coli* to mitigate stress,^[^ ^40^, ^41^, ^42^ ^]^ was observed during imaging of sfGFP expression (Figure S5g, 5 h, Supporting Information) thought to result from a lack of nutrients or oxygen, osmotic stress, or confinement within the hydrogel.^[^ ^43^ ^]^ The potential for Ca^2+^ induced stress was investigated by screening cell viability in response to a range of calcium chloride concentrations in the incubation media of printed constructs using live/dead staining (Figure S7, Supporting Information). Cell filamentation was absent with 10 and 20 mM calcium chloride supplementation but was observed at a calcium chloride concentration of 45 mM (Figure S7d, Supporting Information), indicating that the morphological response was Ca^2+^ concentration dependent, likely due to either osmotic stress or by cell membrane interaction. Confinement effects on morphology within the hydrogel may be minimal, as morphological change was also observed in unconfined suspensions of *E. coli* grown with calcium chloride supplementation (Figure S8, Supporting Information). A filamentous morphology may have utility with respect to improving the yield of intracellular biopolymers and biomass in fermentation technologies,^[^ ^42^ ^]^ and the increased aspect ratio of the cell may also play a role in preventing predation by engulfment.^[^ ^44^ ^]^ For later fabrication and maintenance of catalytic structures, a compromise for calcium ion supplementation between gel integrity and cell morphology was struck at 20 mM.

The benefits of effective segregation of disparate strains for coculture and cascade biosynthesis have been demonstrated.^[^ ^7^, ^45^ ^]^ With functional flexibility in mind, the ability of cell populations to move between bioink filaments was therefore assessed using fluorescence microscopy of lattices printed using distinct microbial inks, containing *E. coli* expressing either sfGFP or mCherry (Figure 3b,e, Figure S1c, S9, Supporting Information). Almost no transfer of either strain was observed at the internal interface between bioinks (Figure 3f), suggesting that the cells could not travel by advection through the bioink's pore network and were limited to localized growth. This entrapment of the majority of the *E. coli* population within the bulk of the gel was assumed to be due either to direct embedding in the walls of the pores or the long distance of travel that would be required to reach the edge owing to its high tortuosity observed by cryoSEM (Figure S10a,b, Supporting Information). After incubation in lysogeny broth (LB), aggregated structures containing a mixed population of sfGFP‐ and mCh‐expressing strains were observed extending into the media away from the gel surface. Despite internal immobilization, improved strategies for the containment of both live cells and genetic material, such as impermeable coatings^[^ ^46^ ^]^ or direct cell‐material bonding,^[^ ^47^ ^]^ would inevitably be required to enable application in nonlaboratory environments. As the function of this ELM arises from intracellular enzyme activity, rather than extracellular release of active catalyst or modification of its environment, robust immobilization of a living bacterial population within the ELM is crucial for sustained use of the ELM in aqueous environments. The rate of cell loss through detachment or death must be less than the cell division rate to maintain a stable or growing population within a reactor, maintaining or improving performance over time and with use.

Interestingly, this effective immobilization was occurring despite the high porosity of the bioink, which was found to be further enhanced under culturing conditions. The pore diameter and diameter variance of *E. coli* laden structures after 24 h of incubation in calcium chloride‐supplemented media were greater than that measured for a cell‐free gel immediately after cross‐linking (mean wall separations of 2.3 ± 2.1 μm and 0.9 ± 0.3 μm, respectively). Significantly, clusters of bacteria were clearly visible within both the porous regions and within the denser wall structures of the bioink (Figure 3g,h, and Figure S10, Supporting Information). As alginate is a reversible, ionically cross‐linked hydrogel, it may be that incubation of the structure in *E. coli* growth media led to swelling along with some dissolution or remodeling of the polymer matrix, causing this increase in pore volume. Despite this, the structures retained their macroscale geometry, key to continuous operation under aqueous flow conditions.

### 2.3 Quiescent OPC Hydrolysis Kinetic Analysis

**Figure 4:**
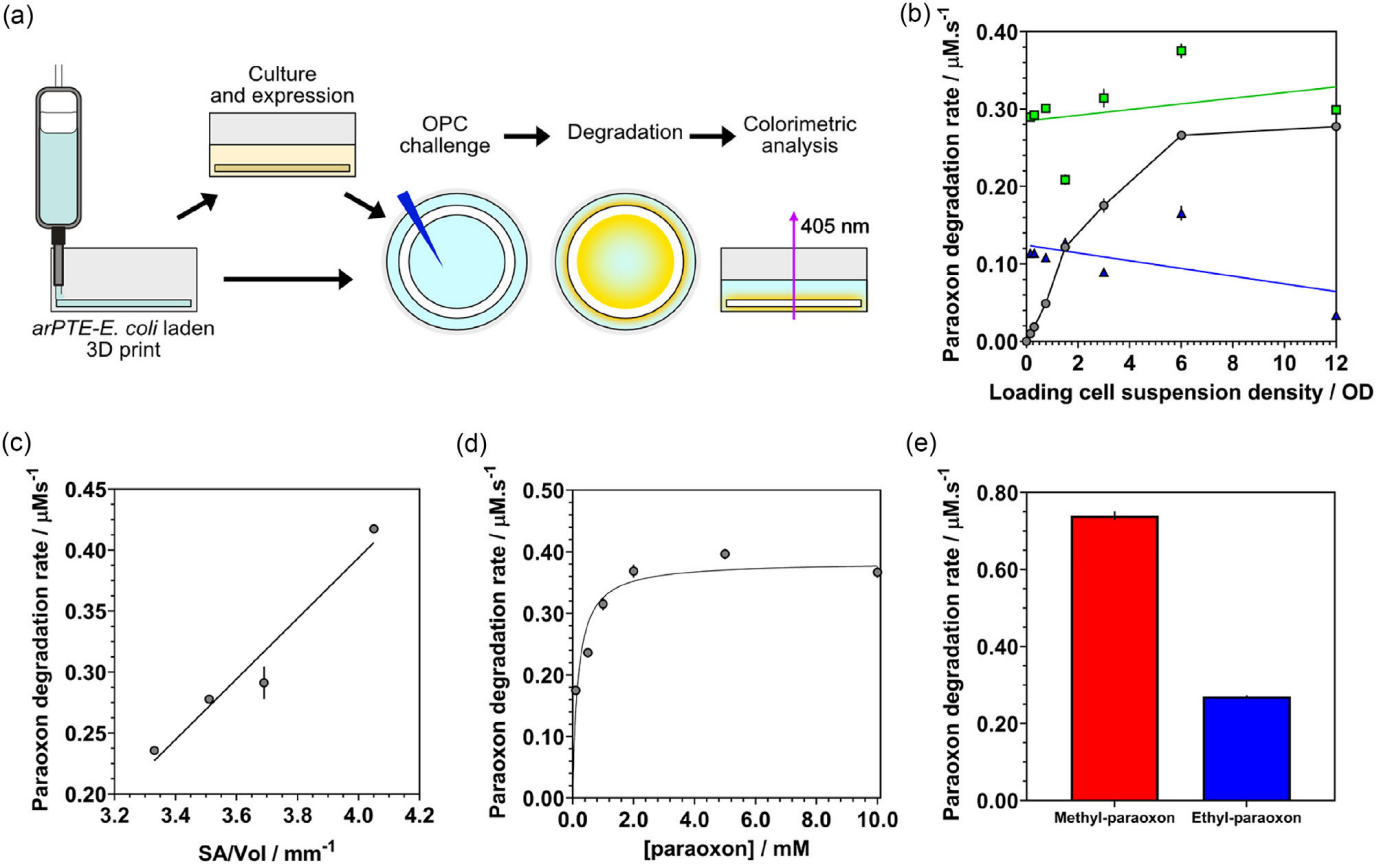
Kinetic characterization of 3D‐printed catalytic microbial ELM rings under quiescent conditions. a) Schematic representation of the preparation of *ar*PTE‐*E. coli* laden ELM rings and their subsequent challenge with OPC under static conditions. b) Initial paraoxon hydrolysis rates for ring constructs with varied initial cell loadings immediately after cross‐linking (‘Day 0’, black circles) and following 24 h of incubation in storage media A with inducer (‘Induced’, green squares, green line, IPTG 1 mM) or repressor (‘Repressed’, blue triangles, blue line, D‐glucose 1w/v%). c) Effect of volume‐specific surface area (SA:Vol) on initial paraoxon hydrolysis rates. d) Dependence of initial paraoxon hydrolysis rates on substrate concentration. Michaelis‐Menten fit. e) Initial hydrolysis rates of methyl‐paraoxon (red) and ethyl‐paraoxon (blue). All error bars are ±1 standard deviation, n = 3 technical repeats; some error bars are too small to display.

Once the support of a sustainable bacterial population confined within the material was confirmed, catalytic function was conveyed to the entrapped cells using inducible expression of the *ar*PTE (Figure S11, Supporting Information). The OPC‐hydrolyzing capability of this ELM was then characterized under quiescent conditions, specifically following the *ar*PTE‐catalyzed hydrolysis of the OPC ethyl‐paraoxon to yellow 4‐nitrophenolate (when buffered to pH 8) and diethyl phosphate (**Figure** **4**a, and Figure S12, Supporting Information).^[^ ^48^ ^]^ Paraoxon degradation rates were determined from the linear region of the production of 4‐nitophenolate, where the ELM was saturated with substrate (Figure S13, S14, Supporting Information). Colorimetric assessment of paraoxon degradation by 3D‐printed ring structures (Figure S1d, S15, Supporting Information) showed a clear increase in rate as cell loading density was increased (‘Day 0’, black circles, Figure 4b), reaching a maximum rate of 0.277 ± 0.05 μM s^−1^ with a loading cell suspension OD of 12. This maximum rate was surpassed by ELM rings of almost all cell loadings that had been induced overnight to overexpress *ar*PTE (‘Induced’, green squares, Figure 4b), with the initial rates observed for lower cell loadings increasing to match this limiting rate around 0.3 μM s^−1^. Crucially, no significant effect of loading cell density was found on degradation rate once overnight growth and induction had been carried out (Figure 4b, green line, slope 0.003 μM s^−1^ OD^−1^, not significantly non‐zero, P = 0.156). Growth overnight in the presence of D‐glucose, to repress *ar*PTE expression, led to a decrease in rate for the higher cell loadings compared to samples assessed immediately after cross‐linking, whilst the performance using cell loadings below an OD of 1.5 increased to just above 0.1 μM s^−1^ (‘Repressed’, blue triangles, Figure 4b, blue line, slope−0.005 μM s^−1^ OD^−1^, significantly non‐zero, P = 0.013). Interestingly, ELMs created with the highest initial cell loading of OD 12 showed a marked decrease in hydrolysis rate when incubated overnight with repressed *ar*PTE expression, whereas high cell density samples, which were induced, exhibited only a slightly higher rate compared to Day 0 ELM samples. The rates observed for the repressed samples after overnight incubation suggest that there was a maximum sustainable cell capacity matching a loading suspension OD of ≈1.5, as the performance of all loading cell densities increased or decreased to a similar level around 0.1 μM s^−1^. This limiting performance is clearly shown when comparing the fold‐change in rate of the induced or repressed samples versus their Day 0 equivalent constructs. Where only constructs loaded with cell densities below an OD of 1.5 show a large increase over a nonincubated sample (Figure S16, Supporting Information).

The observed plateau in initial rates for the quiescent ELM reactors at a cell loading OD of 6 and 12 indicated that the system had either reached a geometrically imposed mass transport limit or that higher cell numbers were not successfully immobilized in the gel due to losses during cross‐linking, as suggested by viability staining. To investigate this proposed mass transport limit, printed ring constructs, fabricated using an undiluted OD 1.8 suspension of *ar*PTE‐*E. coli*, were divided with a scalpel to generate new surfaces while maintaining equal volume before challenge with OPC (Figure S15, Supporting Information). Significantly, observation of a clear linear relationship between measured initial paraoxon degradation rates and the volume‐specific surface area corroborated the hypothesis that mass transport into the ELM was limiting performance (Figure 4c). For the construct with the largest volume‐specific surface area, a rate of 0.417 ± 0.003 μM s^−1^ was recorded, above the previously measured limiting rate for undivided rings with the same cell loading.

The OD 1.8 suspension loaded rings were then challenged with a range of concentrations of ethyl‐paraoxon to understand the behavior of the immobilized whole‐cell system compared to free aqueous *ar*PTE. The resulting rates closely matched a Michaelis‐Menten (MM) model with substrate–concentration independence at high substrate concentrations (Figure 4d). The *K*_m_ of the ELM was significantly higher than aqueous *ar*PTE (0.18 mM vs 0.05 mM, respectively; Table S3; Figure S17, Supporting Information), indicating a considerable decrease in enzyme–substrate interactions. Despite likely containing a higher concentration of enzyme, the *V*_lim_ of the ELM was found to be lower than that for the aqueous enzyme (0.38 μM s^−1^ vs 0.66 μM s^−1^, respectively). This suggests that there was significant impairment of the reaction due to the mass transfer of substrate or product through the ELM. This is especially marked, as enzyme efficiency could be expected to be greater in vivo compared to in solution, as suggested by the *k*_cat_ increase recorded for aqueous enzyme in the presence of a bovine serum albumin (BSA) crowder, often used to mimic intracellular conditions (Figure S17a,b, Supporting Information). Furthermore, the ELM‐catalyzed rate of hydrolysis of methyl‐paraoxon was more than double that recorded for ethyl‐paraoxon (0.74 ± 0.01 μM s^−1^ and 0.271 ± 0.003 μM s^−1^, respectively; Figure 4e). This is less than the near fourfold difference reported between the enzyme concentration‐normalized limiting rates (*k*_cat_) for aqueous *ar*PTE when degrading methyl‐ and ethyl‐paraoxon,^[^ ^49^ ^]^ suggesting that the more hydrophilic methyl‐paraoxon was being impeded more than ethyl‐paraoxon during the path of transport into and out of the ELM (Log K_OW_ of methyl‐ and ethyl‐paraoxon 0.98 and 1.97, respectively).^[^ ^50^ ^]^ The hydrophilicity of methyl‐paraoxon may have resulted in more frequent or stronger interactions with the alginate matrix, something not often considered within hydrogel diffusion models,^[^ ^51^ ^]^ or its traversal of the cell membrane may have been slower than ethyl‐paraoxon as has previously been reported.^[^ ^33^ ^]^

### 2.4 3D‐Printed Continuous Flow Reactor Application

**Figure 5:**
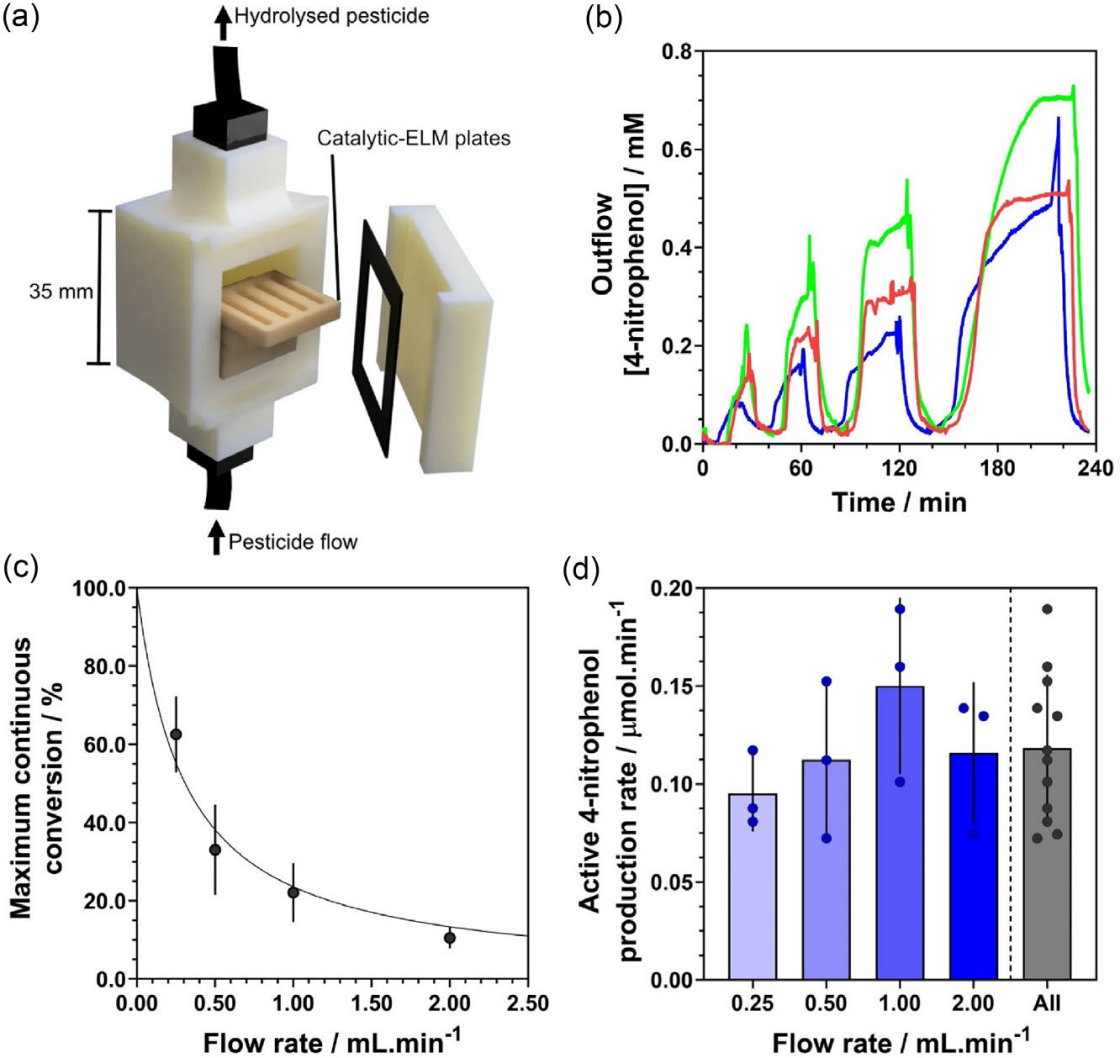
Incorporation of 3D‐printed catalytic microbial ELM structures into a flow reactor for continuous OPC hydrolysis. a) CAD rendering of the reactor assembly. b) Outflow 4‐nitrophenolate concentration during cycled application of paraoxon to the reactor with intermittent buffer washes (troughs), three experimental repeats shown. c) Maximum continuous paraoxon‐hydrolysis performance as a percentage of applied paraoxon for each flow rate. Reciprocal relationship fit using a nonlinear least squares fitting process according to Equation 1 (*C* = *k*/(*F *+ *k*/*C*_max_), *k* = 30.87 % mL min^−1^, *C*_max_ = 100%, r^2^ = 0.94). d) Average 4‐nitrophenolate production per minute during the paraoxon application stage at 0.25, 0.50, 1.00, and 2.00 mL min^−1^, along with the average for all flow rates tested, no significance between any pair of flow rates (one‐way ANOVA, Brown‐Forsythe, P > 0.05). Error bars are ±1 standard deviation, n = 3 experimental repeats.

Making use of the described ELM's 3D printability, self‐supporting porous structure, and effective immobilization of a catalytically active living *E. coli* population, a flow reactor system capable of continuous OPC hydrolysis was fabricated using a combination of stereolithographic (SLA) 3D printing and extrusion bioprinting (**Figure** **5**a, Figure S18, S19, S20, Supporting Information). Four consecutive applications of ethyl‐paraoxon (1 mM) into the reactor at decreasing flow rates were separated by buffer washes at 2 mL min^−1^, with 4‐nitrophenolate output concentration measured downstream of the reactor (Figure 5b). Whilst constant 4‐nitrophenolate output was achieved at a flow rate of 0.25 mL min^−1^, a steady state was not reached during application of paraoxon at 2.00, 1.00, or 0.50 mL min^−1^, suggesting that the following efficiencies are underestimates of the steady state potential of the system. Peak continuous conversion efficiencies of 10.5, 22.1, 33.0, and 62.5% were recorded during cycled application of OPC substrate solution at flow rates of 2.00, 1.00, 0.50, and 0.25 mL min^−1^, respectively (Figure 5c). These data fit well to a reciprocal relationship between continuous conversion and flow rate (Equation (1), k = 30.87% mL min^−1^, *C*_max_ = 100%, nonlinear least squares fit, r^2^ = 0.94)

(1) $$C=\frac{k}{F+\frac{k}{C_{\text{max}}}}$$

where *C* is the continuous conversion efficiency (%), the fitting parameter *k* characterizes the relationship between conversion efficiency and flow rate, *F* is the flow rate (mL min^−1^), and *C*_max_ is the maximum theoretical conversion (%). Whilst the overall efficiency of the reactor was strongly flow rate dependent, this fit also suggests that the reactor operated at a constant absolute conversion rate, independent of flow rate. This is most clearly seen when normalizing for the volume of substrate solution being applied (Figure 5d). The system diluted the 4‐nitrophenolate output from the ELM, likely operating at a mass transport‐limited rate with no significant difference in active 4‐nitrophenol production rate as flow rate was varied (Figure S21, Supporting Information). Moreover, no significant loss in function during cycled OPC application and intermittent buffer washes was observed, suggesting there was limited loss of catalyst or active cells during flow. Variation in both the maximum conversion rate achieved at each flow rate and the acceleration of the conversion rate after the addition of paraoxon to the reactor was observed between repeats (Figure 5b; red, green, blue). This is thought to result from temperature variation.

The large channels of the 3D‐printed lattice plates and their alignment with the inlet and outlet ports of pseudofractal flow distributors^[^ ^52^, ^53^ ^]^ minimized pressure drop across the reactor, preventing damage to the ELM plates (Figure S20c, Supporting Information). This reduction in pressure drop across a device is crucial for hydrogel‐based immobilized whole‐cell reactors. As the stiffness of the alginate matrix was low and the containment of the bacterial population was maintained only through the structural integrity of the bulk gel, damage from impinging jets of solution that extended into the bulk material would have led to large losses of cell population and subsequently a drop in catalytic performance. This could have also compromised the recoverability of the ELM's performance through incubation, due to a loss of suitable microbial habitat.

### 2.5 Reaction and Diffusion Processes within the ELM

The above quiescent analysis, application within a 3D‐printed flow reactor device, and the identification of yellow 4‐nitrophenolate product accumulation within the flow reactor ELM plates (Figure S20b,c, Supporting Information), confirmed that mass transfer was a defining limitation of this material. Identification of the active catalyst's position relative to the boundaries and interfaces in the reactor was therefore crucial for future reactor design and optimization. Accordingly, a sfGFP fusion enzyme, sfGFP–*ar*PTE was engineered to investigate the enzyme's location within the ELM via fluorescence microscopy. Correct folding of the purified chimera was confirmed both computationally, using Alphafold (sequence and structure given in Table S4 and Figure S22, Supporting Information, respectively),^[^ ^54^ ^]^ and experimentally, using circular dichroism (CD) (Figure S23, Supporting Information).^[^ ^55^ ^]^ Time‐course widefield fluorescence microscopy of the solution–ELM interface of rings laden with *E. coli* expressing sfGFP–*ar*PTE was then used to monitor the position of the sfGFP–*ar*PTE (Figure S1e, S24e, Supporting Information). The OPC‐hydrolyzing activity of the sfGFP–*ar*PTE fusion was confirmed by the generation of a blue, fluorescent concentration gradient directed away from and into the ELM when challenged with Coumaphos (Figure S24, Supporting Information), which produces chlorferon after hydrolysis (Figure S25, Supporting Information).^[^ ^34^ ^]^ Blue fluorescence emerging from outside of the printed construct would have indicated the presence of free catalyst diffusing around and away from the ELM.

**Figure 6:**
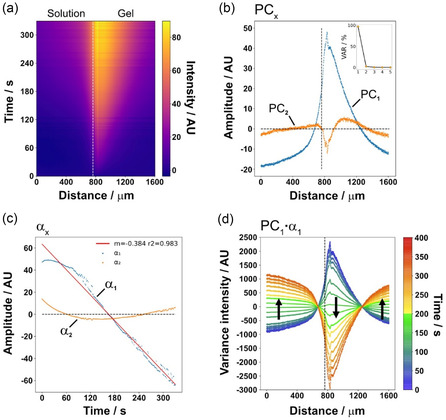
PCA of continuous fluorescence imaging data of reaction diffusion at the ELM–solution interface, with diffusion constrained to one interface. a) Surface‐axis‐averaged chlorferon intensity over time as a function of distance along an axis perpendicular to the ELM–solution interface (white dashed line represents interfacial x‐position, with solution to the left and ELM to the right, identified by microscopy). b) The first two principal components (PC_1_, blue and PC_2_, orange) at all locations, (vertical black dashed line represents interface). Inset: Scree‐plot showing relative proportion of variance accounted for by the first five components in order of proportion. c) The magnitude of the coefficient for the first two components as a function of time (*α*_1_, blue; *α*_2_, orange). d) Linear combination of principal component one (PC_1_) with its coefficient (*α*_1_), giving PC_1_ *α*_1_, displayed at discrete timepoints (vertical black dashed line represents interface). Color to timepoint mapping given alongside the figure.

To investigate the concentration field formed by the reaction and diffusion process occurring after Coumaphos addition, a widefield fluorescence microscopy time‐course was recorded across the gel–solution interface of a geometrically simplified (rectangular) sfGFP–*ar*PTE‐*E. coli* laden ELM sheet (Figure S1f, S26, Supporting Information). The kymograph resulting from this time‐course imaging showed a clear peak of chlorferon production occurring ≈100 μm into the hydrogel sheet (to the right of the white dashed line at 760 μm), with a diffusion front emanating from this interface both into and away from the ELM (**Figure** **6**a). Dimensional reduction was carried out using spatiotemporal PCA. After normalizing the dataset to have a mean of 0 and standard deviation of 1, PCA represented the intensity data (*I(x, t)*) as a linear combination of components, each a product of a function in *x* and *t*, along with the mean of the data (*Ī*) (Equation (2)).

(2) $$I\left(x,t\right)=\;{\text{PC}}_{1}\left(x\right){\;\alpha }_{1}\left(t\right)+{\text{PC}}_{2}\left(x\right){\;\alpha }_{2}\left(t\right)\;+\ldots \;\ldots +{\text{ PC}}_{n}\left(x\right){\;\alpha }_{n}\left(t\right)\;+\;\bar{I}$$

where each component is the product of a shape mode (PC_*n*_(*x*)) and a temporal coefficient (*α*_*n*_(*t*)). This generated a series of principal components that were ranked according to the magnitude of variance they accounted for. From this (inset, Figure 6b), it was found that over 90% of the variance could be explained using only the first principal component, allowing the others to be discarded without significant loss of information. Looking at the spatial form of this first principal component (PC_1_, blue line, Figure 6b), it could be seen that peak relative variance was occurring just below the ELM surface, with below average (<0) relative variance displayed in the substrate reservoir (left of the dashed line) and deeper within the ELM sheet (beyond ≈ 1300 μm). Interestingly, whilst the spatial modes were nonmonotonic, the coefficient of PC_1_ through time (*α*_1_, blue, Figure 6c) was approximately linear (r^2^ = 0.983), displaying an inversion of behavior relative to the mean ≈150 s after substrate addition. When linearly combined to form the first principal component pair (PC_1_ *α*_1_, Figure 6d), it could be seen that peak acceleration of chlorferon production (positive relative variance) was occurring just within the ELM sheet at early timepoints (blue–green, Figure 6d), reaching steady state after 150 s as the relative variance became negative. This suggested that changes in production rates were greater elsewhere along the spatial axis at these later timepoints. Initially, in the solution phase (to the left of the dashed line), a diffusion gradient was set up close to the ELM surface. Further into the solution, negative relative variance could be seen, suggesting that chlorferon diffusion into the solution was being outstripped by production within the ELM. Again, this inverted at roughly 150 s, suggesting the reservoir was beginning to saturate.

Given that the product must first leave an immobilized cell by traversing the membrane to diffuse, variance is dominated at early timepoints by localized chlorferon production, present only at locations that contain active cells and have received substrate. The product is essentially trapped within these areas, leading to a local increase in fluorescence. Once the material is operating at or near its steady state, i.e., the rate of production of chlorferon no longer changes within the ELM, then variance is dominated by the diffusion of chlorferon that has made it out of the cells (yellow–red, Figure 6d), leading to the positive relative variance seen in both the deep ELM phase (beyond ≈ 1300 μm) and within the solution. The higher positivity in the solution phase is the result of the increased diffusion rate through the solution.

In the linear combination plot (Figure 6d), the points where the mean‐relative variance intensity was maintained at zero are potentially useful as limits for use within catalytic reactor design. If designing a flow reactor system, such as the one discussed above, the root at ≈1300 mm may suggest a suitable maximum depth for this catalytic ELM of 500 μm. Additionally, the inversion time of 150 s may suggest a suitable minimum duration to allow a reactor to reach steady state and give time for product diffusion to occur before the substrate solution exits a reactor. For example, if the residence time of a theoretical plug of substrate solution passing the ELM is less than the transition at 150 s, then any ELM deeper than the root at ≈500 mm would not be releasing product during the passage of that plug of solution. Constraining the reactor wall depth to ≈500 μm could ensure efficient use of the material in the case that only one ELM face is exposed to the substrate. These parameters are material, catalyst, and substrate specific. Comparison of different reactive material systems using this analytical method could uncover practical relations between the ELM composition, material microstructure and geometry, and the desired reaction, enabling flow reactor optimization.

## 3 Conclusion

Using 3D bioprinting, we have brought together a highly biocompatible porous biomaterial and genetically engineered microbial catalysts to create readily fabricable living microreactors capable of robust continuous degradation of OPCs. We have demonstrated that the ELM microreactors can continuously hydrolyze OPCs at 60% efficiency (molar product output vs molar OPC input, per second) at a flow rate of 0.25 mL min^−1^ and withstand multiple cycles of pesticide injection. The reactor was found to be limited by mass transport, which demonstrates the importance of access to the high volume‐specific‐surface‐area structures afforded by 3D printing. PCA of temporal fluorescence imaging data of the reaction diffusion process occurring within the ELM revealed potential key design parameters (e.g., gel wall depth or substrate reactor residence time), suitable for reactor optimization, which could be used to develop a putative methodology for the assessment of new catalytic ELMs. This will be critical if the multiplexed dynamics of the substrate, material, living cell population, enzymatic catalyst, and product in flow are to be understood.

Flow path customization and the potential for modularity through long‐term compartmentalized coculture remain key potential benefits of 3D printable ELMs. At slow flow rates with sufficient interplate mixing, enzyme cascade reactions could be set up through replacement of duplicate ELM plates with those expressing enzymes matching a reaction pathway. Coexpression of enzyme variants, such as those designed for the degradation of a suite of OPCs,^[^ ^56^ ^]^ could also widen the substrate specificity of this type of device. In addition to catalytic modularity, nonreactive modules could also be included. Given the inclusion of suitable chemical, pH, or temperature sensitive gene circuitry within immobilized bacteria,^[^ ^10^, ^57^, ^58^ ^]^ externally readable sensing outputs could be generated based on conditions that affect reactor catalytic performance. A multifunctional biosensor reactor ELM device capable of producing fluorescent signals^[^ ^59^ ^]^ in response to OPC concentrations, coupled to PTE expression, would represent a marked improvement over current pesticide and CWA detection and removal strategies. The confined growth shown within this material, combined with the capability to fabricate wide channels, may also reduce the impact of overgrowth and clogging compared to a surface‐immobilized, packed‐bed system.^[^ ^60^ ^]^ The integrity of the bioink could also be reinforced to ensure minimal cell loss during flow, for example, using dual ion cross‐linking,^[^ ^61^ ^]^ thereby improving the longevity of the reactor. Significantly, the 3D bioprinted engineered living microreactors reported here are field‐fabricable; they are comprised of inexpensive, shippable, and storable materials that do not require a logistical cold chain, paired with a relatively simple assembly method that depends only on small 3D printers. Contingent upon the hazards of a particular application, the internal living material component could also be readily disassembled and replaced using calcium chelators such as ethylenediaminetetraacetic acid.^[^ ^62^ ^]^

The 3D printability of this ELM enabled effective characterization of both the behavior of an encapsulated microbial cell population as well as the performance of the ELM as a biocatalyst under quiescent and flow conditions. However, the full utility of 3D printability was not leveraged with respect to flow control and surface area maximization within the context of the flow reactor. Further work on the design of continuous flow ELM bioreactors may look to use 3D printing of materials such as the templated porous alginate described above, to fabricate higher specific‐surface‐area structures and nonmoldable geometries. For example, gyroids, 3D lattices, and channels, or structures designed to suit application‐specific geometric and size constraints, e.g., for integration into existing drainage systems or portable bioreactor devices. Use of novel 3D printable double networks and nanocomposite hydrogels,^[^ ^63^, ^64^ ^]^ along with the extensive suite of synthetic biology tools now available for catalyst localization and genetic regulation, could lead to a step change in the performance of these reactors through minimization of the substrate and product mass transport pathways. Further research into creating environmentally robust, catalytically efficient microbial strains as model ELM chassis, along with suitable biocontainment systems, will also be necessary for the wide‐ranging application of such ELMs for bioremediation. As these challenges are addressed, the application of 3D bioprinting for ELM design and fabrication is set to bring about new sustainable solutions to some of the most pressing contemporary bioremediation challenges.

## 4 Experimental Section

4.1

4.1.1

##### Strains and Plasmids

The strains and plasmids used within this work and their sources are described in Table 1, 2, Supporting Information, respectively.

##### E. coli Culture

*E. coli* strains picked from streaked colonies (agar, 15 mg L^−1^ in LB, 25 g L^−1^; carbenicillin, 50 g mL^−1^) were cultured overnight shaking at 37 °C in LB (15 mL, 25 g L^−1^) supplemented with D‐glucose (10 mg mL^−1^) and carbenicillin (50 μg mL^−1^). OD at 600 nm (OD_600_) was measured after 18 h of growth. Cell suspensions were either centrifuged at 3500 × *g* for 15 min, followed by removal of excess supernatant, and then resuspended to concentrate the cells, or were diluted with sterile LB to reach the desired OD_600_ before incorporation into the bioink.

##### Bioink Creation

A modified protocol adapted from J. Armstrong et al.^[^ ^36^ ^]^ was used. For a 5 mL final gel: sodium alginate powder (325 mg, UV sterilized for 1 hr) and carbenicillin (5 μL, 50 mg mL^−1^) were mixed into an overnight LB cell culture suspension (2.25 mL, varied OD_600_) using a dual asymmetric centrifuge (DAC, Speedmixer, 3500 rpm, 5 min). Calcium chloride solution (CaCl_2 (aq)_, 1 mL, 75.0 mM, sterile) was then mixed into the gel by DAC (3500 rpm, 2 min). Cold Poloxamer 407 hydrogel (1.75 mL, 400 mg mL^−1^ in deionized water, autoclaved and homogenized by DAC, 5 °C) was then mixed into the precross‐linked alginate gel by DAC (3500 rpm, 2 min). This gave a final ink composition of 6 wt% alginate, 13 wt% Poloxamer 407, and 15 mM CaCl_2_. Bioinks were used for printing immediately after creation.

##### Bioprinting

Digital 3D geometries created using Fusion360 (Autodesk) were exported as STL files and sliced using Heartware (Cellink) with the Slic3r plugin to generate their respective gcode. Bioinks were loaded from 5 mL syringes into 3 mL pneumatically driven syringe barrels using a double female Luer lock connector to minimize bubble creation. An Inkredible bioprinter (Cellink) modified through the addition of a heated copper print‐bed was used to extrude bioink. The custom heated bed was set to maintain a temperature of 42 °C, and 5 °C above the estimated LCST of the ink formulation to account for heat loss and slow heat transfer through a microplate to the gel. An 18 G nozzle (internal diameter 838 μm) was used for all printing. After printing, all alginate/Poloxamer constructs were submerged in calcium chloride solution (CaCl_2 (aq)_, 100.0 mM, sterile) for 30 min (unless otherwise stated) to cross‐link the alginate polymer and wash the Poloxamer phase from the gel. For overnight growth, prints were incubated at 37 °C shaking in storage media A (LB, 25 g L^−1^; carbenicillin, 50 μg mL^−1^; calcium chloride, 20 mM) with protein expression achieved through the addition of L‐arabinose (10 mg mL^−1^) for sfGFP and IPTG (1 mM) for mCherry, *ar*PTE, and *ar*PTE‐sfGFP. Storage media A was supplemented with D‐glucose (10 mg mL^−1^) to repress protein expression.

##### Printing Mass Variability Assessment

A cylindrical model was sliced using either a concentric or rectilinear infill pattern. Sets of cylinders made using each code (height 4 mm, diameter 11 mm) were printed into 9 cm petri dishes and cross‐linked using calcium chloride (100 mM, submerged) overnight. Surface water was removed once using a paper towel and their masses were measured (wet mass). All cylinders were desiccated overnight at room temperature under vacuum and then their masses were measured (dry mass).

##### CryoSEM of Cross‐Linked Prints

For cell‐free samples, samples were taken immediately after cross‐linking; for cell‐containing samples, samples were incubated overnight in storage media **A** at 37 °C, shaking, and then rinsed before cryoSEM sample prep. Before imaging, all samples were frozen by submersion in a liquid nitrogen slurry under vacuum and then fractured by shear force. Water at the fracture surface was sublimated over 3 min at −92 °C under vacuum, with a localized condenser plate to prevent ice deposition on the sample. After sublimation, the top surface of the sample was sputter‐coated with gold/palladium to prevent charging during image acquisition.

##### 4‐Nitrophenolate Diffusion Coefficient Determination

A hydrogel containing 1 mg mL^−1^ of 4‐nitrophenol was created through the addition of 2.22 mg mL^−1^ 4‐nitrophenol to the LB used to dissolve the alginate fraction of the bioink. Cylinders of bioink (4 mm high, 11 mm diameter) and alginate hydrogel control (6.5 w/v% in LB supplemented with 4‐nitrophenol, 1 mg mL^−1^) were cast and cross‐linked in calcium chloride solution (submerged, 100 mM; supplemented with 4‐nitrophenol, 1 mg mL^−1^) for 1 h at 37 °C and then stored overnight in the same solution at 4 °C. 3D‐printed resin gel traps were inserted into a six‐well plate containing reaction buffer B (HEPES, 30 mM; CoCl_2_, 100 μM, pH 8, 10 mL per well). The 4‐nitrophenol‐loaded cylinders were returned to room temperature and their surface dried briefly using a paper towel before being transferred to the trap of a buffer‐containing well for immediate measurement of 4‐nitrophenolate loss. Absorbance was recorded at 405 nm through the center of the well for 10 min with a 4 s measurement interval at 22 °C using a plate reader (BioTek Synergy Neo 2). The height and diameter of each cylinder were then measured. The concentration of 4‐nitrophenolate in the well was calculated using the Beer‐Lambert law, assuming an extinction coefficient of 15 550 cm^−1^ M^−1^ at 405 nm and pH 8. The path length was automatically determined by comparison of a test absorbance at 977 nm to a reference absorbance at 900 nm. Using this data, the diffusion coefficient was determined using a semi‐infinite slab approximation.^[^ ^65^ ^]^ Briefly, assuming the 4‐nitrophenolate concentration within the hydrogel was constant at early timepoints, molecular flux out of the hydrogel structure is given by Equation (3).

(3) $$j=\left(c_{\text{solution}}-c_{\text{matrix}}\right)\cdot {\left(D_{\text{matrix}}/\pi t\right)}^{1/2}$$

where *j* is the molecular flux, and at initial timepoints, *c*_solution_ is assumed to be 0, *c*_matrix_ is the loading concentration of 4‐nitrophenolate (1 mg.mL^−1^), and *D*_matrix_ is the diffusion coefficient of 4‐nitrophenolate through the hydrogel matrix. This was integrated to give Equation (4).

(4) $$c_{\text{solution}}=c_{\text{matrix}}\cdot \left(\frac{A_{\text{matrix}}}{V_{\text{bath}}}\right)\cdot 2\cdot \left(t\cdot D_{\text{matrix}}/\pi \right)1/2$$

where *A*_matrix_ is the hydrogel surface area and *V*_bath_ is the volume of the surrounding solution. *D*_matrix_ was then calculated from the gradient of the linear relationship of *c*_solution_ versus *t* ^1/2^.

##### Fluorescent Spectroscopy Measurements of sfGFP and mCherry Expression

Droplets of bioink (sfGFP‐ or mCherry‐*E. coli* OD_600_ 1.5 loading, repressed) were extruded into a 96‐well plate and cross‐linked in calcium chloride solution (100 mM, 100 μL per well, 10 min). The cross‐linking solution was aspirated by pipette and replaced with inducing or repressing storage media A. The droplets were incubated for 24 h at 37 °C with fluorescence intensity of sfGFP (excitation 479 nm, emission 520 nm) and mCherry (excitation 579 nm, emission 616 nm) measured through the center of each well every 2 min using a plate reader (BioTek Synergy Neo 2). After 24 h of incubation, the media was replaced with fresh inducing or repressing storage media A and the droplets were incubated for a further 24 h with continued fluorescence measurement as above.

##### Viability Determination

Single‐layer discs (*ar*PTE‐*E. coli* OD 0.5, 1.5, and 6.0; 15 mm diameter, one layer) were printed (18 G^1/2^” needle) for viability determination at 0 h, after printing and cross‐linking, and 24 h, after printing, cross‐linking, and incubation in storage media A. The prints were washed once with sterile saline solution (1 mL, 0.9 w/v%, NaCl_(aq)_) and then stained in SYTO9 (10 μM)/propidium iodide (60 μM) in sterile saline solution (0.5 mL, 0.9 w/v%, NaCl_(aq)_) for 45 min at room temperature in the dark. The staining solution was removed with a pipette, and the print was washed twice with sterile saline (2 × 1 mL, 0.9 w/v%, NaCl_(aq)_) and the saline removed. The gel discs were then imaged using confocal fluorescence microscopy within the 24‐well plate (Leica SP8 confocal laser scanning microscope). Viability was calculated using pixel counting after background subtraction and thresholding to sum only the signal from cell size and shaped objects. Live cell count was determined as (green pixels–red pixels)/green pixels.

##### Visualization of Encapsulated Cell Growth

Single‐layer discs of *E. coli*‐sfGFP‐laden bioink were imaged using widefield fluorescence microscopy (Leica DMI6000 inverted epifluorescence microscope) immediately after cross‐linking and after 24 h of shaking incubation in storage media A at 37 °C. Directionality of colonies within the printed fibers was assessed using the ImageJ Directionality plugin.^[^ ^66^ ^]^

##### Dual‐Ink sfGFP and mCherry Strain Localization

Dual‐ink lattices (alternating layers) were printed into six‐well plates using dual extrusion of sfGFP‐expressing and mCherry‐expressing bioinks (sfGFP‐*E. coli* and mCherry‐*E. coli* OD_600_ 1.5 loading). After cross‐linking, the lattices were incubated overnight at 37 °C in storage media A with the addition of L‐arabinose (1 w/v%) to induce sfGFP expression and IPTG (1 mM) to induce mCherry expression. Lattices were washed with sterile saline solution (3 mL, 0.9w/v%, NaCl_(aq)_) and the saline removed before imaging using confocal fluorescence microscopy in the six‐well plate.

##### Calcium Chloride Tolerance Screening

Printed cell laden ring constructs were incubated overnight at 37 °C in LB media (0.5 mL, 25 g/L, carbenicillin at 100 μg mL^−1^) supplemented with varying concentrations of calcium chloride (10, 15, 20, 25, 30, 35, and 40 mM). Syto9/propidium iodide staining was carried out as described above to image the cell populations by widefield fluorescence microscopy.

##### Quiescent Bioreactor Cell Density Variation with Incubation and Induction

Six‐well rings (*ar*PTE‐*E. coli* varied OD_600_ loading, major radius 31 mm, minor radius 27 mm, height 0.84 mm) were printed directly into a six‐well plate and cross‐linked by submersion in calcium chloride solution (CaCl_2,_ 3 mL per well, 100 mM) for 30 min. This allowed for scatter free measurement of absorbance through the center of the well. The rings were transferred to a fresh six‐well plate and a 3D printed plastic ring was inserted into each well to prevent movement of the ELM across the well (Figure S15, Supporting Information). The effect of initial cell loadings (loading cell suspension OD 0.00, 0.15, 0.30, 0.75, 1.50, 3.00, 6.00, and 12.00) on the initial rates of ethyl‐paraoxon degradation was tested immediately after cross‐linking (‘Day 0’) through measurement of 405 nm absorbance. The samples were submerged in reaction buffer B (2.940 mL) and the reaction initiated through the addition of ethyl‐paraoxon (60 μL, 50 mM in 2‐propanol, and final concentration 1 mM) to each well. Immediately after the addition of ethyl‐paraoxon, absorbance at 405 nm was recorded through the center of each well using a plate reader (BioTek Synergy Neo 2) with a 30 s read interval until a plateau for all wells was reached. Initial rates were determined from the rate of change of absorbance, where this change was linear, ranging up to 30 min of reaction. Six‐well ring structures with the above cell loadings (*ar*PTE‐*E. coli)* were incubated overnight in inducing or repressing storage media A to create ‘Induced’ and ‘Repressed’ samples, respectively. After incubation, the media was aspirated off and the rings were washed once in reaction buffer B (2 mL). The rings were submerged in reaction buffer B (2.940 mL) and the reaction was initiated by the addition of ethyl‐paraoxon (60 μL, 50 mM in 2‐propanol, final concentration 1 mM). Initial rates were determined colorimetrically, as above.

##### Quiescent Bioreactor Volume‐Specific Surface Area Variation

Six‐well rings were prepared as described above (OD_600_ 1.8 loading). Intact rings were used as a minimal volume‐specific surface area construct, and gel constructs with varying volume‐specific surface area were created by cutting the rings into four, eight, or 16 equal pieces; these samples had volume‐specific surface areas of 2.14, 2.35, 2.56, and 2.98, respectively. The rings (and divided rings with resin spacers) were submerged in reaction buffer B (2.940 mL) and the reaction initiated by the addition of ethyl‐paraoxon (60μL, 50 mM in 2‐propanol, final concentration 1 mM). Initial rates were determined colorimetrically, as above.

##### Quiescent Bioreactor Substrate Concentration Variation

Six‐well rings were prepared as described above (OD_600_ 1.8 loading). Varied paraoxon concentrations (0.1, 0.5, 1.0, 2.0, 5.0, and 10.0 mM) were assessed through the addition of ethyl‐paraoxon (50 mM in 2‐propanol; 6, 30, 60, 120, 300, and 600 μL) to rings submerged in respective volumes of reaction buffer B (2.994, 2.970, 2.940, 2.880, 2.700, and 2.400 mL). Initial rates were determined colorimetrically, as above.

##### Quiescent Bioreactor Substrate Variation

Six‐well rings were prepared as described above (OD_600_ 1.8 loading). After cross‐linking, the samples were submerged in reaction buffer B (HEPES, 30 mM; CoCl_2_, 100μM, pH 8, 2.940 mL) and the reaction was initiated through the addition of ethyl‐ or methyl‐paraoxon (60 μL, 50 mM in 2‐propanol, final concentration 1 mM) to each well. Initial rates were determined colorimetrically, as above.

##### ELM Flow‐Lattice Fabrication

Gcode for flow reactor lattices was manually written to prevent the generation of unwanted travel moves. The lattice plates were ≈20 mm across, six layers high (≈5 mm), with a double filament wall thickness of ≈2 mm, printed using an 18 G ½” needle at a pressure of ≈80 kPa, traveling at 10 mm s^−1^ during extrusion.

##### Reactor Design and Fabrication

Unidirectional lattice plates (*ar*PTE‐*E. coli* OD_600_ 6 loading) were printed, cross‐linked (1 h, submerged in CaCl_2_ (100 mM)), and incubated in storage media A at 37 °C shaking overnight with IPTG induction (1 mM). After 24 h, the plates were rinsed with sterile saline (0.9w/v%) to remove surface growth and were stacked into a square cross‐sectioned reactor (six lattices per reactor, ≈9 mL internal volume, 20 × 20 mm) designed using Fusion360 (Autodesk) and 3D printed using a Form2 printer (Formlabs). Threaded Delrin connectors and PVC tubing were used to attach the inlet and outlet to a syringe pump (AL‐1000, World Precision Instruments) and quartz flow cell (1 mm pathlength, 300 μL, Starna Scientific Ltd), respectively.

##### Cycled Ethyl‐Paraoxon Challenge with Flow Rate Variation

Sterile saline solution was flown through the reactor to displace the air inside (20 mL, 0.9 w/v%, 4.00 mL min^−1^). The reactor was then equilibrated with reaction buffer B (25 mL, 2.00 mL min^−1^). A solution of ethyl‐paraoxon (1 mM in reaction buffer B, 20 mL, 2.00 mL min^−1^) was then driven through the reactor using a syringe pump, followed by buffer solution to wash the system (B, 40 mL, 2.00 mL min^−1^). This was then repeated using the same reactor system at paraoxon flow rates of 1.00, 0.50, and 0.25 mL min^−1^ with buffer washes in between each paraoxon application (B, 40 mL, 2.00 mL min^−1^). Absorbance was recorded downstream of the reactor within the flow cell at 405 nm using an Agilent Cary 60 UV/Vis Spectrophotometer. A scan interval of 4.8 s was used. Before disassembly, concentrated bleach was flown through the reactor (40 mL, manual rate) followed by deionized water (40 mL, manual rate).

##### 3D SLA Printing of Inserts and Flow Reactor Casing

All custom parts and inserts (for quiescent OPC degradation and reaction diffusion imaging) and the flow reactor casing were printed on a Form2 using Formlabs’ Clear V4 resin, washed twice in iso‐propylalcohol for 15 min, and left to cure in daylight for at least 1 day before use.

##### sfGFP‐arPTE Fusion Plasmid Assembly

Genes for sfGFP and *ar*PTE were cloned into an empty pET14b plasmid using Gibson Assembly^[^ ^67^ ^]^ and the resulting vector transformed by heat shock into chemically competent *E. coli* (DH5*α*) for plasmid stabilization. The plasmid was extracted and purified using a GeneJET Plasmid Miniprep kit (Thermofisher) and sequenced by Sanger sequencing (Eurofins). The sequenced plasmid was then transformed by heat shock into chemically competent *E. coli* (BL21 DE3) for protein expression and ELM incorporation.

##### sfGFP‐arPTE Expression

A cold induction protocol was followed to express sfGFP‐*ar*PTE within BL21 (DE3). LB media (10 mL, 25 g L^−1^) supplemented with D‐glucose (5w/v%) was inoculated directly with a glycerol stock of sfGFP‐*ar*PTE‐*E. coli* and incubated shaking overnight at 37 °C. The starter culture (7.5 mL) was transferred to a sterile solution of terrific broth (1 L, 47.6 g L^−1^, glycerol, 0.8 v/v%, autoclaved) supplemented with D‐glucose (0.75 g L^−1^, autoclaved) and cobalt chloride (CoCl_2_, 100 μM, autoclaved) and grown shaking at 37 °C to an OD_600_ of 0.5. The suspension was then induced with IPTG (1 mM) and incubated shaking at 20 °C for two days. The resulting suspension was centrifuged at 4000 × *g* for 20 min, the supernatant discarded, and the pellet stored at −20 °C before purification.

##### sfGFP‐arPTE Purification

After IPTG‐induced expression of the fusion protein, sfGFP‐arPTE was purified using an immobilized metal affinity column (IMAC) and size exclusion chromatography (SEC). A frozen pellet of induced sfGFP‐*ar*PTE‐*E. coli* was thawed and resuspended in lysis buffer (NaCl, 1M; HEPES, 30 mM; CoCl_2_, 100 μM; imidazole, 50 mM; pH 8) with benzonase (25 U mL^−1^) and PMSF (phenylmethylsulfonylfluoride, 1 mM) and lysed by sonication (60 Hz, 2 s on, 2 s off, 3 min × 4, 1 min rest between cycles). The lysed cells were centrifuged at 10 000 × *g* for 30 min at 4 °C. The supernatant was then purified by IMAC (XK 16 packed with 15 mL nickel‐charged nitriloacetic acid (NTA) coupled to Sepharose beads, equilibrated with degassed lysis buffer). Supernatant was loaded onto the column at 2 mL min^−1^, and the column was washed with lysis buffer. A gradient elution was then carried out: 100% lysis buffer to 100 % elution buffer (NaCl, 1M; HEPES, 30 mM; CoCl_2_, 100 μM; Imidazole, 500 mM; pH 8, degassed), 45 min, 2 mL min^−1^. The eluent was transferred directly onto a SEC column for further purification (HiLoad 26/600 Superdex 200 pg (Cytiva), equilibrated with buffer solution (NaCl, 1 M; HEPES, 30 mM; CoCl_2_, 100 μM; pH 8)). Further equilibration buffer was applied at 2 mL min^−1^, and absorbance was monitored in line at 280 and 487 nm, with 12 fractions collected across observed peaks. Fraction contents were identified by sodium dodecyl sulfate‐polyacrylamide gel electrophoresis.

##### sfGFP‐arPTE Structural Characterization

Fractions selected from appropriate SEC elution peak were dialyzed overnight into CD buffer (K_2_PO_4_, 10 mM; Na_2_SO_4_, 50 mM; pH 8). The resulting concentrated solution was diluted to ≈2 mg mL^−1^, filtered (0.22 μm, Sartorious), and equilibrated within a 1 mm path length quartz cuvette at 25 °C for 5 min. A full spectral scan was recorded using a Jasco J‐1500 CD spectrophotometer (range, 260–190 nm; data pitch, 1 nm; direct integration time, 4 s; bandwidth, 2 nm; scanning speed, 5 nm min^−1^; accumulations, 3; temperature, 25 °C). The resulting data were input into the Beta structure selection (BeStSel) spectral analysis software for secondary structure estimation.^[^ ^68^ ^]^ CD at 222 nm was also recorded during a temperature ramp (start temp., 25 °C; end temp., 95 °C; interval, 0.5 °C; equilibration time per temp., 12 s; direct integration time, 4 s; bandwidth, 1 nm; wavelength, 222 nm).

##### sfGFP‐arPTE Hydrolysis Activity Characterization

To initially determine the activity of the new fusion, 24‐well ring prints of standard bioink, loaded with sfGFP‐*ar*PTE‐*E. coli* BL21(DE3) at OD 1.8 (grown overnight without D‐glucose repression), were submerged after cross‐linking in 2 mL of reaction buffer B and challenged with a solution of Coumaphos (1 mM final concentration). Widefield fluorescence microscopy was used to image this addition immediately at 0.25 s intervals for ≈5 min.

##### Diffusion Time Series Imaging

Rectangular constructs (20 × 5 mm, 2 layers) were printed (18 G^1/2^” needle) using standard bioink (sfGFP‐*ar*PTE‐*E. coli*, OD_600_ 6, induced in suspension for 4 h at 37 °C by the addition of 1 mM IPTG after overnight growth) and then cross‐linked for 30 min in CaCl_2_ (100 mM, submerged). Individual cross‐linked constructs were transferred to six‐well plates and individually trapped underneath 3D‐printed resin inserts. Time‐lapse imaging of the gel edge (a single plane at the half height of the gel) was carried out with a 5 s image interval. A solution of Coumaphos (1 mM) in reaction buffer B was added to the reservoir a few seconds into the time‐lapse. The formation of chlorferon, the product of the hydrolysis of Coumaphos, was imaged through excitation of fluorescence with a wavelength of 355 nm and emission intensity was measured at 460 nm.

##### Image Processing and PCA

Each time series of fluorescence images was averaged along the axis of the ELM–solution interface and baseline corrected to t_0_ (the point of substrate addition), generating trimmed datasets up to 330 s long, saved as.csv files. These datasets were used as the input for PCA. Briefly, to generate the spatial modes (PC_*x*_), the 2D data were first normalized ((data‐mean)/standard deviation) and then its correlation matrix was produced. The eigenvectors and values from this matrix represent the component and its magnitude, respectively. To generate the temporal coefficients (*α*_*x*_) corresponding to the spatial modes, the normalized data were transposed, and a correlation matrix was generated from this transposed data. The eigenvectors and values from this matrix describe the temporal coefficients. The resulting components were output in graphical form. A linear combination of the first spatial mode (PC_1_) and its temporal coefficient (*α*_1_) was then produced to display the evolution of this mode through time (PC_1_·*α*_1_).

##### Statistical Analysis

Data are presented as mean ± SD unless otherwise stated in figure caption. For the analysis of the significance of the diffusivity of 4‐nitrophenolate through the bioink and alginate, a sample size of 6 was used, using an unpaired two‐tailed *t*‐test with Welch's correction, and significance was considered as P < 0.05. For analysis of significance between active 4‐nitrophenol production at varied flow rates (Figure 5d, Figure S21, Supporting Information), n = 3 experimental repeats, a one‐way ANOVA (Brown‐Forsythe) with multiple comparisons (between all pairs of flow rates) was carried out with significance considered as P < 0.05. All statistical analysis was performed using Graphpad Prism.

## Conflict of Interest

The authors declare no conflict of interest.

## Supporting information

Supplementary Material

## Data Availability

The data that support the findings of this study are available from the corresponding author upon reasonable request.
