# Supplementary material for: 3D Bioprinted Engineered Living Microreactors for Continuous Organophosphorus Compound Degradation
Source: Small Sci. 2025 Sep 4;5(11):2500226. doi: 10.1002/smsc.202500226 (PMC12622513; doi:10.1002/smsc.202500226)
Supplement: Supplementary file 1 — Supplementary Material [file SMSC-5-2500226-s001.pdf]

Supporting Information

3D Bioprinted Engineered Living Microreactors for Continuous Organophosphorus Compound Degradation

*Mark R. Shannon, Graham J. Day, Hermes Bloomfield-Gadêlha, Valeska P. Ting, and Adam W. Perriman**

Contents

E. COLI STRAINS AND PLASMIDS 5

BIOINK STRUCTURE AND 3D PRINTING 7

EXPERIMENTAL 3D PRINT DIVERSITY 7

3D PRINT MASS CONSISTENCY 8

BIOINK DIFFUSION COEFFICIENT DETERMINATION 9

FLUORESCENCE IMAGING AND SEM 10

LIVE/DEAD IMAGING 10

SFGFP WIDEFIELD FLUORESCENCE IMAGING – ONE DAY INCUBATION 11

FIBRE-ALIGNED GROWTH 12

CA^{2+} SUPPLEMENTATION – SYTO9/PROPI^{-} STAINING IN GEL 13

CA^{2+} SUPPLEMENTATION IN SUSPENSION 14

CONFOCAL FLUORESCENCE – SFGFP MCHERRY DUAL INK LATTICES 15

CRYOSEM 16

OPC DEGRADATION 17

*AR*PTE STRUCTURE 17

MECHANISM OF PTE CATALYSED PARAOXON HYDROLYSIS 17

GENERATION OF 6 WELL RING STRUCTURES FOR QUIESCENT OPC HYDROLYSIS. 18

COLORIMETRY DATA FOR QUIESCENT 6 WAELL RING REACTIORS WITH CELL DENSITY VARIATION AND OVERNIGHT GROWTH WITH INDUCER OR REPRESSOR. 19

COLORIMETRY DATA FOR QUIESCENT 6 WELL RING REACTIORS WITH VARIED SA/VOL, VARIED SUBSTRATE HYDROPHOBICITY, AND VARIED SUBSTRATE CONCENTRATION. 20

OPC HYDROLYSIS WITH VARIED CELL DENSITY FOLLOWED BY INDUCTION OR REPRESSION OF *AR*PTE EXPRESSION. ... 21

MM KINETIC CHARACTERISATION OF ELM AND AQUEOUS *AR*PTE 22

FLOW REACTOR CASING 22

FLOW REACTOR PRACTICAL SETUP 24

One way ANOVA (Brown-Forsythe) for active 4-nitrophenol production at varied flow rates. 25
sfGFP-*ar*PTE fusion creation and characterisation 26
sfGFP-*ar*PTE Alphafold predicted structure 26
sfGFP-*ar*PTE circular dichroism 27
sfGFP-*ar*PTE functional imaging 28
Mechanism of PTE catalysed Coumaphos hydrolysis 29
Reaction-diffusion imaging experimental sequence 30

E. coli strains and plasmids

*Table **1**: Bacterial strains used within this work with respective description and source.*

Strains	Description and phenotype	Source
Escherichia coli DH5α	General cloning strain with mutations in the recombinase *recA* and endonuclease *endA1* to improve plasmid stability and DNA yield, respectively. Blue/white colour screening also possible with *lacZ*ΔM15.	New England Biolabs
E. coli BL21(DE3)	Protein expression strain lacking the Lon and OmpT proteases, containing the phage T7 RNA polymerase under IPTG inducible promoter.	New England Biolabs
E. coli-sfGFP	*E. coli* BL21(DE3) transformed with pBAD-sfGFP	This work
E. coli-mCh	*E. coli* BL21(DE3) transformed with pET45b-mCherry-ST3	This work
E. coli-*ar*PTE	*E. coli* BL21(DE3) transformed with pMCS1-*ar*PTE	This work
E. coli-sfGFP-*ar*PTE	*E. coli* BL21(DE3) transformed with pET14b-sfGFP-*ar*PTE	This work

*Table **2**: Plasmids used within this work with respective description and source.*

Plasmid	Description	Source
pBAD-sfGFP	L-Arabinose inducible expression plasmid; with *sfGFP* under control of ARA promoter and the *araC* gene for expression of AraC repressor, along with *ampR* conferring resistance to carbenicillin.	In group
pET45b-mCherry-ST3	IPTG inducible expression plasmid; with *mCherry-ST3* under control of T7 promoter and *lacI* gene for expression of LacI repressor, along with *ampR* conferring resistance to carbenicillin.	In group
pMCS1-*ar*PTE	IPTG inducible expression plasmid; with *arPTE* under control of T7 promoter along with *ampR* conferring resistance to carbenicillin.	In group
pET14b-sfGFP-*ar*PTE	IPTG inducible expression plasmid; with *sfGFP-arPTE* under control of T7 promoter along with *ampR* conferring resistance to carbenicillin.	This work

Bioink structure and 3D printing

Experimental 3D print diversity

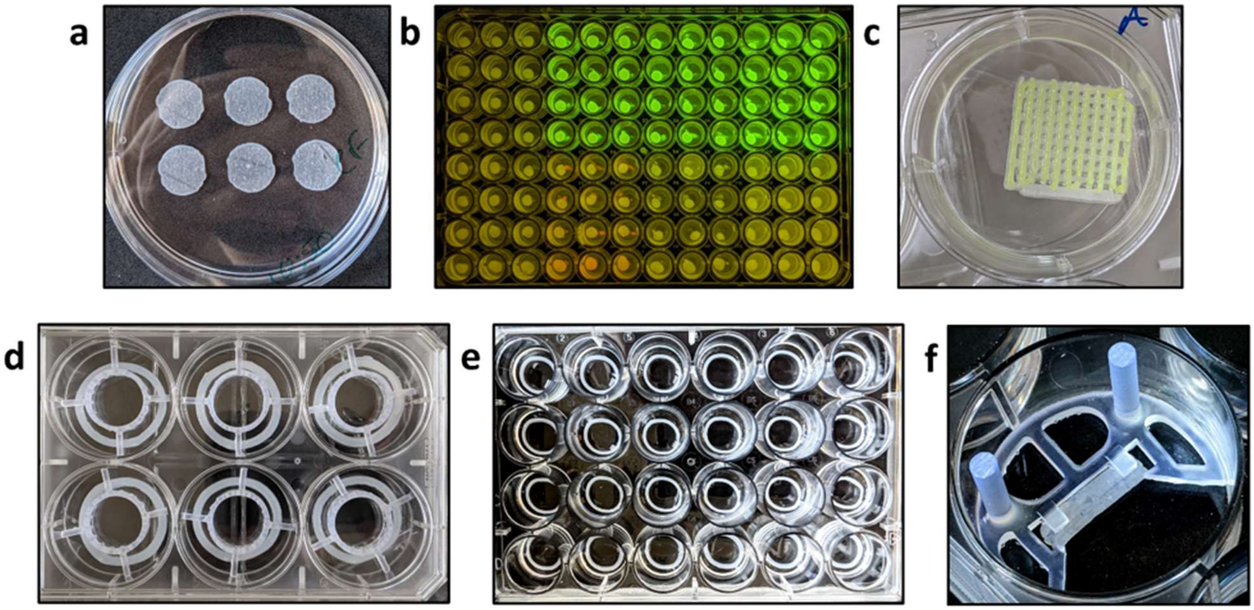

*Figure **1**: Exemplary images of 3D printed objects used throughout this work, for both fluorescence microscopy of immobilised microbial populations and functional OPC degradation assays in quiescent and flow systems. All printed using an 18 G needle. **a**) Single layer 15 mm diameter discs used for confocal and widefield imaging of sfGFP fluorescence and live/dead (SYTO9/propidium iodide) stained samples. **b**) 96-well droplet prints containing either E. coli-sfGFP or E. coli-mCherry, with blue light transillumination, used for investigating protein expression dynamics by chemical induction with L-arabinose or IPTG, respectively, through measurement of fluorescence intensity. **c**) Dual extruded 2 cm square lattice, lower layer printed with E. coli-mCherry, upper layer printed with E. coli-sfGFP. In this case induced with only L-arabinose for sfGFP expression, showing selective control of protein expression between two cell populations immobilised in the same lattice, used to image neighbouring populations of cells to determine the extent of immobilisation in the gel. **d**) Single layer six-well rings laden with E. coli-arPTE, trapped underneath 3D printed resin inserts for colorimetric measurement of quiescent ethyl-paraoxon degradation. **e**) E. coli-sfGFP-arPTE laden 24-well rings used for preliminary timelapse widefield fluorescence imaging of Coumaphos degradation and chlorferon diffusion. **f**) E. coli-sfGFP-arPTE laden 20 x 4 mm single double layer cuboid trapped underneath a 3D printed resin insert, used to image reaction and diffusion of Coumaphos into and away from the living material.*

3D print mass consistency

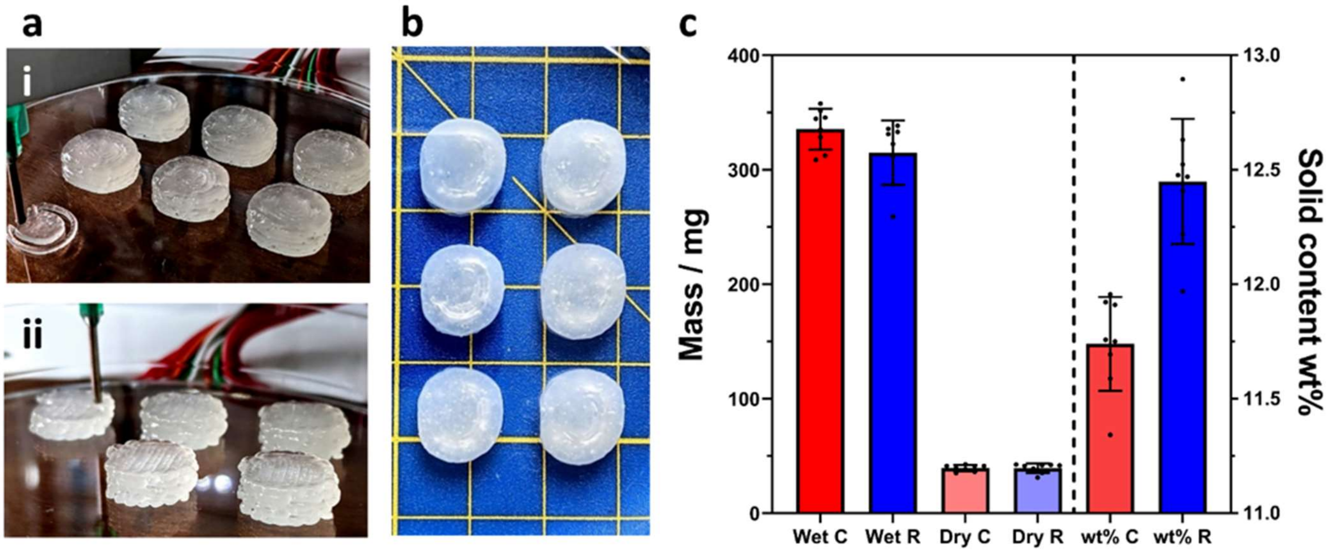

*Figure **2**: Comparison of the inter-print mass consistency for concentric and rectilinear infill patterns. **a**) Pre-crosslinked cylindrical structures during the extrusion printing process; **i**) concentric infill pattern, **ii**) rectilinear infill pattern, cylinder diameters approximately 11 mm, and 4 layers, ~3 mm high. **b**) Top down view of six fully crosslinked concentric cylinders, grid squares 10 mm. **c**) Wet (after crosslinking) and Dry (after 24-hour desiccation) masses for cylinders printed using concentric (C) and rectilinear (R) infill patterns (left of division), along with the dry to wet weight percentages (solid content) for both printing patterns (right of division) giving an estimate for the final polymer wt% after crosslinking has occurred.*

Bioink diffusion coefficient determination

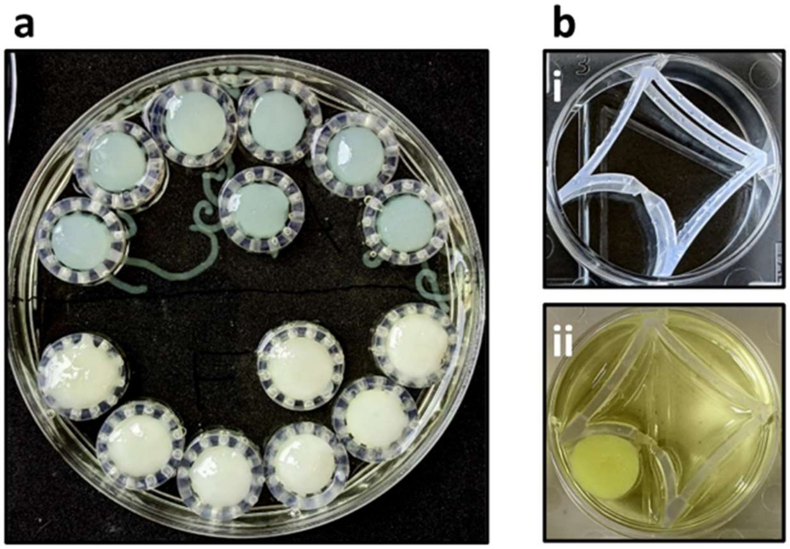

*Figure **3**: **a**) Moulded alginate (top, grey) and bioink gels (bottom, yellowish grey), supplemented with 4-nitrophenol (1 mg/mL), crosslinking in a bath of $CaCl_2$ (100mM) also supplemented with 4-nitrophenol (1 mg/mL). Protrusion of the alginate samples from the moulds has occurred due to swelling of the hydrogel. **b**) **i**) 3D printed plastic inserts used to trap the gel cylinders during continuous measurement of 4-nitrophenolate release, printed using a Form2 printer and V4 clear resin (Formlabs). **b**) **ii**) Endpoint photo of a gel cylinder in a resin trap submerged in HEPES/$CoCl_2$ (30 mM/100 μM, pH 8, 10 mL) after measurement of 4-nitrophenolate release.*

Fluorescence imaging and SEM

Live/dead imaging

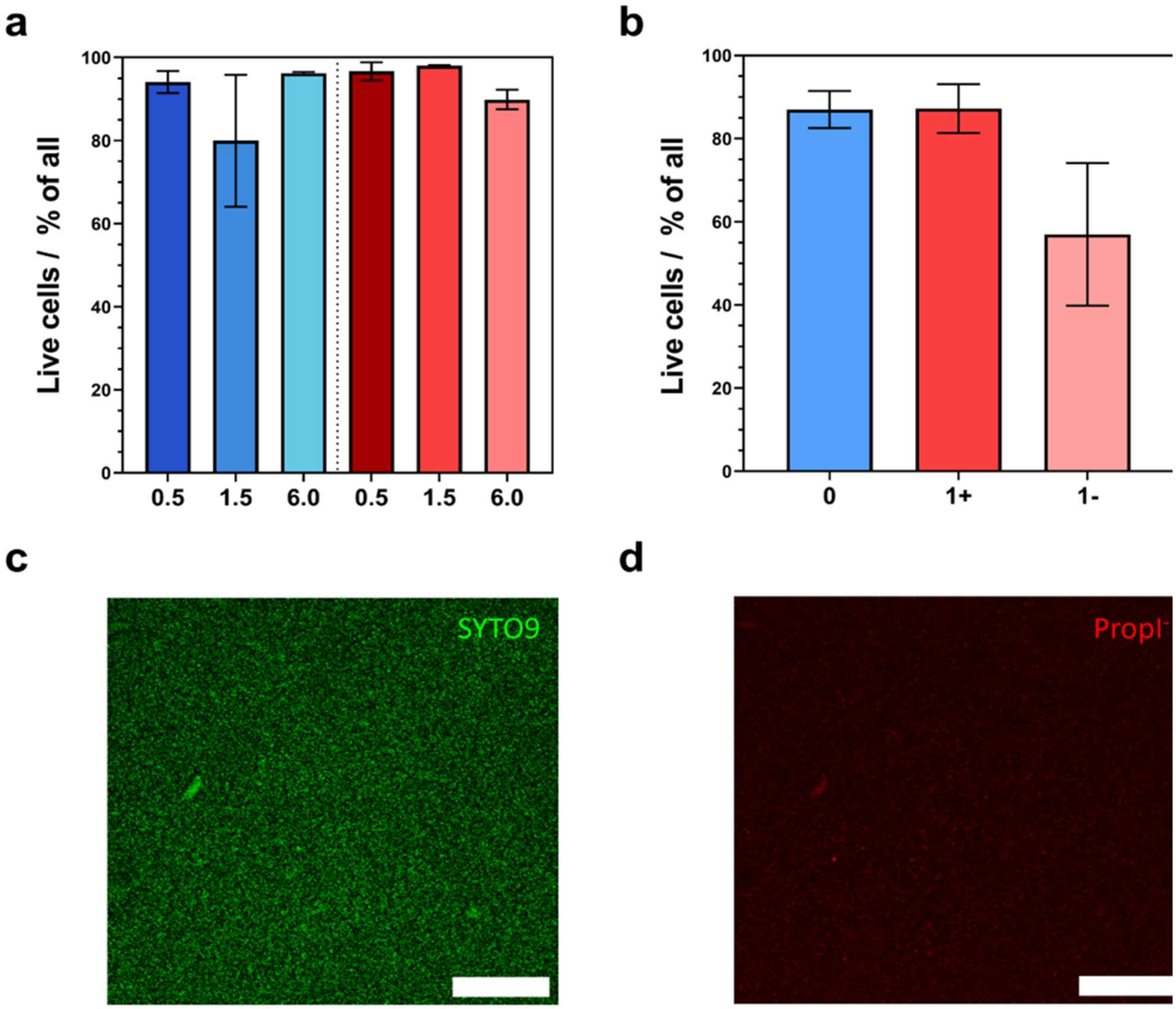

*Figure **4**: Quantitation of viability and cell coverage from fluorescent confocal laser scanning microscopy images of SYTO9 (live, green)/Propidium iodide (PropI⁻, dead, red) stained E. coli within ELM discs at initial loading suspensions concentrated to an OD_{600} of 0.5, 1.5, and 6.0. **a**) Viability measured as the percentage live cells, (green pixels – red pixels) / green pixels for each cell loading at 0 (blue, left) and 24 (red, right) hours after crosslinking the printed ELM discs. **b**) Change in viability of E. coli before (0) and 24 hours after induction of protein expression (1 mM IPTG, 1+) or repression (1wt% D-glucose, 1-). **c**, **d**) Representative live (**c**, green) and dead (**d**, red) maximum z-projections of the E. coli laden ELM. Gel depth was ~500 µm.*

sfGFP widefield fluorescence imaging – one day incubation

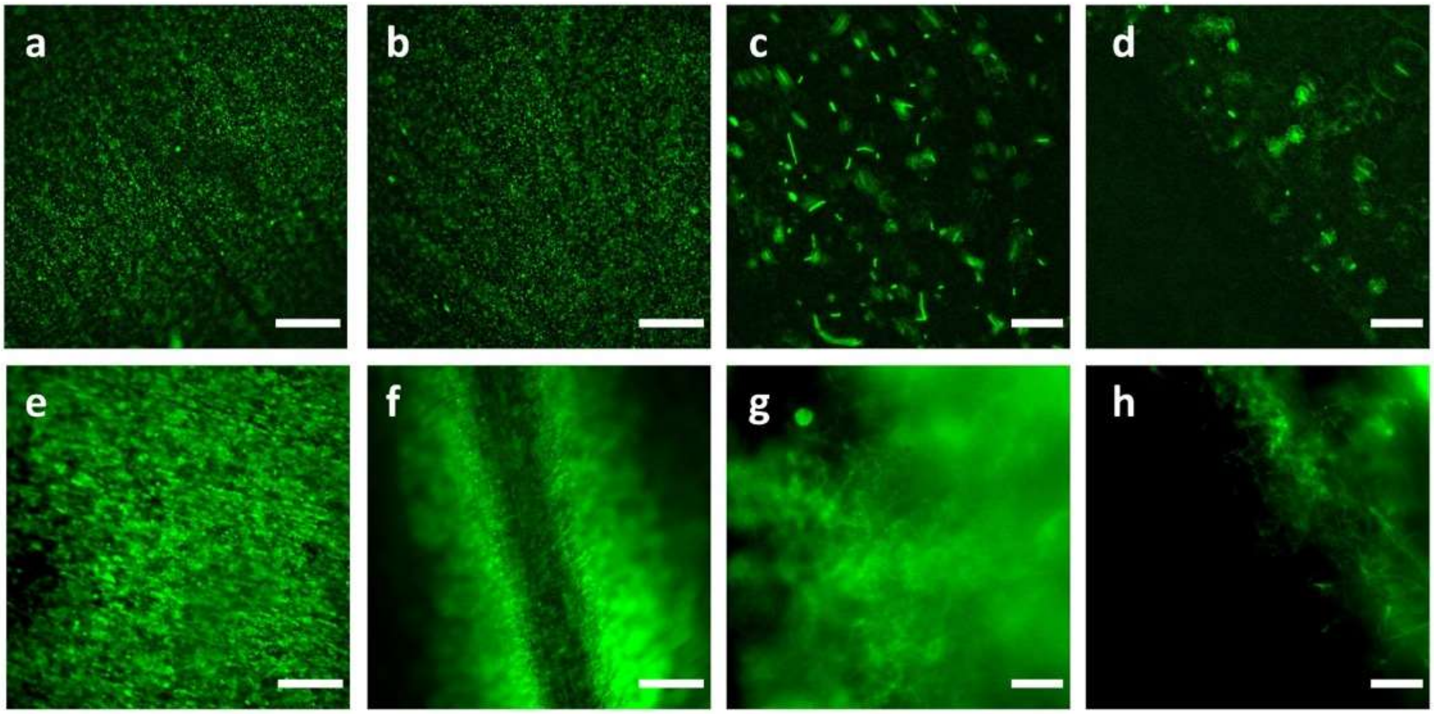

*Figure **5**: Widefield fluorescence imaging of E. coli-sfGFP (green) laden, crosslinked prints both immediately after crosslinking, displaying homogenously dispersed individual and short chains of bacteria: **a**), **b**), **c**) **d**), and after 24 hours of incubation shaking at 37 °C in storage media **A**, showing the formation of biofilm like structures and microcolonies: **e, f**), along with the presence of filamentous E. coli: **g**), **h**). **a**, **b**) A vertical filament (right hand side) crosses the diagonally aligned filaments, possibly generated by a printhead travel move dragging through the print , scale bars 500 μm. **c**) Higher magnification image of planktonic cells within the bulk of the gel showing short chains of cells resulting from the polar cell division of E. coli, scale bar 50 μm. **d**) Higher magnification image of the edge of the gel, showing the presence of a dispersed population of cells retained close to the surface of the gel (gel edge roughly curving from top left corner to bottom right), scale bar 50 μm. **e**) Diagonally oriented filaments, scale bar 500 μm. **f**) Crossing filament, showing an increased density of cells surrounding the filament relative to its centre, potentially suggesting nutrient diffusion limitations into the centre of the gel, and that surface growth (out of plane with the centre of the filament imaged here) is well supported, scale bar 500 μm. **g**) Higher magnification image of the biofilm-like structure within the bulk of the gel, showing the presence of interconnected network of cells, scale bar 50 μm. **h**) Higher magnification image of the edge of the gel, showing that the initially dispersed bacteria present at the gel surface have grown into continuous filamentous structures (>50 long) extending away from the gel, potentially suggesting that they are under stress, scale bar 50 μm.*

Fibre-aligned growth

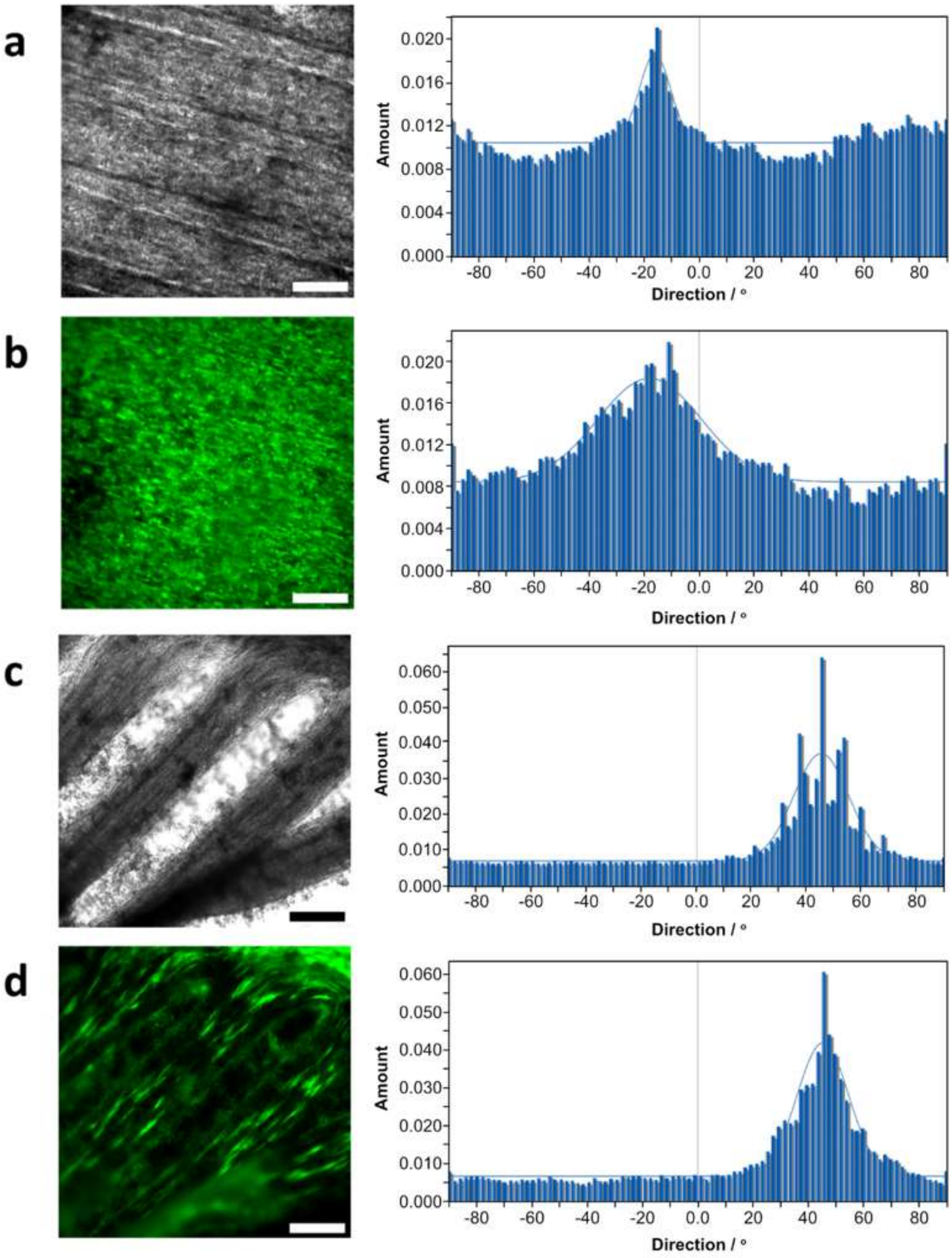

*Figure **6**: Alignment of sfGFP expressing E. coli microcolonies (green) with printed fibre direction after incubation. **a**) Brightfield image of horizontally fused parallel fibres in a single layer, major direction -16.03°. **b**) sfGFP fluorescence signal of view **a**, showing embedded colonies of E. coli, with major direction -18.02°. **c**) Brightfield image of near parallel fibres (dark) with fibre width spacing (white areas), major direction 45.37°. **d**) sfGFP fluorescence signal of view **c**, showing embedded colonies of E. coli along with bridging growth between filaments, major direction 45.25°. Directionality calculated using ImageJ directionality plugin. Scale bars 500 µm.*

Ca^{2+} supplementation – Syto9/PropI^{-} staining in gel

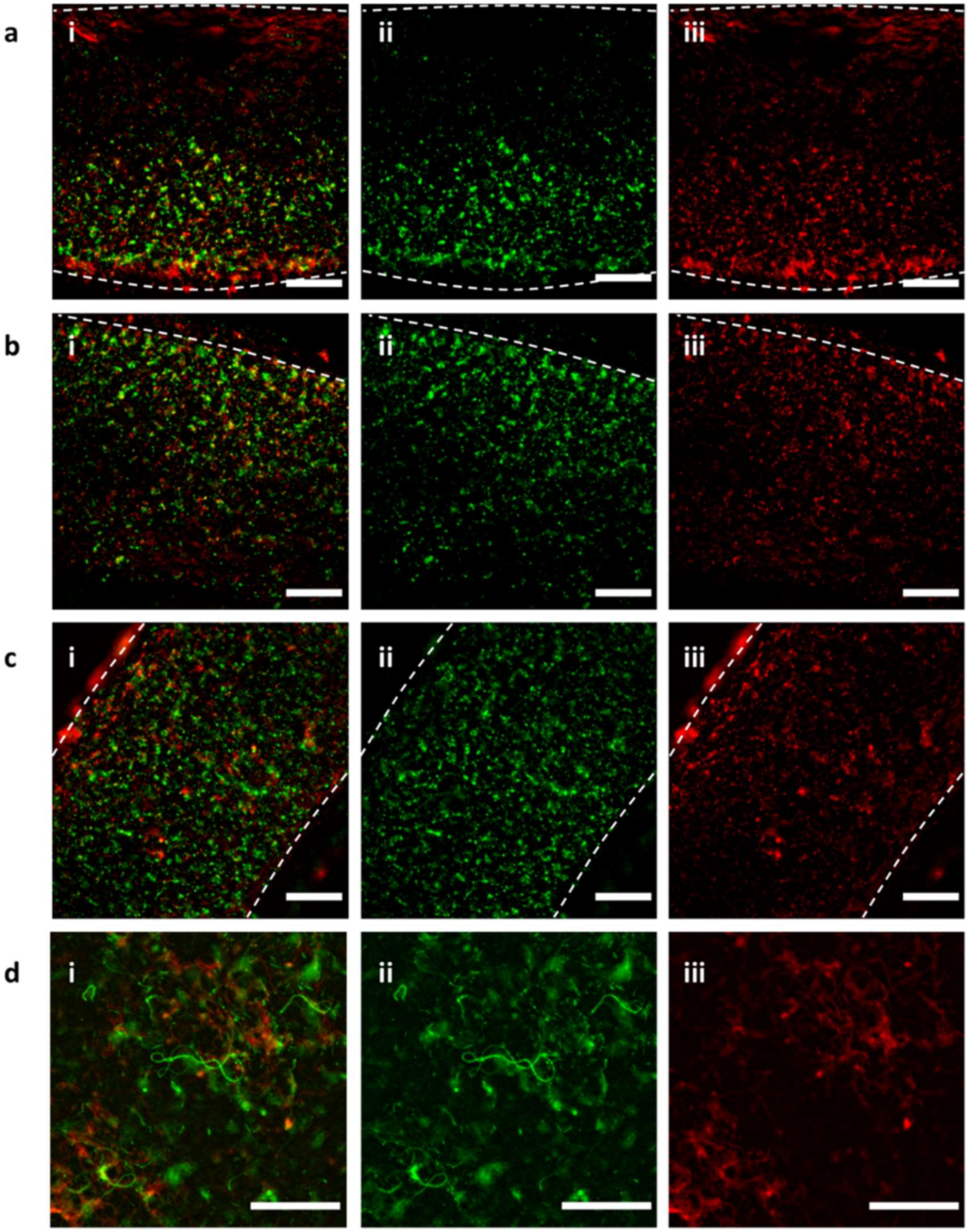

*Figure **7**: Maximum Z projections of widefield fluorescence images of Live/dead (SYTO9 (green)/propidium iodide (red)) stained gels loaded with arPTE expressing E. coli BL21(DE3) incubated overnight at 37 °C in LB/carbenicillin with varying $CaCl_2$ concentrations. Populations of bacteria are visible throughout the gel, with a significant proportion of live cells in all conditions. **a**) 10 mM $CaCl_2$ supplement; **i**) Overlay of SYTO9 and propidium iodide signal, **ii**) SYTO9 live signal, **iii**) propidium iodide dead signal. **b**) 20 mM $CaCl_2$ supplement; **i**) Overlay of SYTO9 and propidium iodide signal, **ii**) SYTO9 live signal, **iii**) propidium iodide dead signal. **c**) 45 mM $CaCl_2$ supplement; **i**) Overlay of SYTO9 and propidium iodide signal, **ii**) SYTO9 live signal, **iii**) propidium iodide dead signal. Gel boundaries marked with dotted lines, scale bars 250 μm. **d**) High magnification single-plane widefield fluorescence image of the 45 mM $CaCl_2$ supplemented gel showing presence of live filamentous bacteria within; **i**) Overlay of SYTO9 and propidium iodide signal, **ii**) SYTO9 live signal, **iii**) propidium iodide dead signal. Scale bars 100 μm.*

Ca^{2+} supplementation in suspension

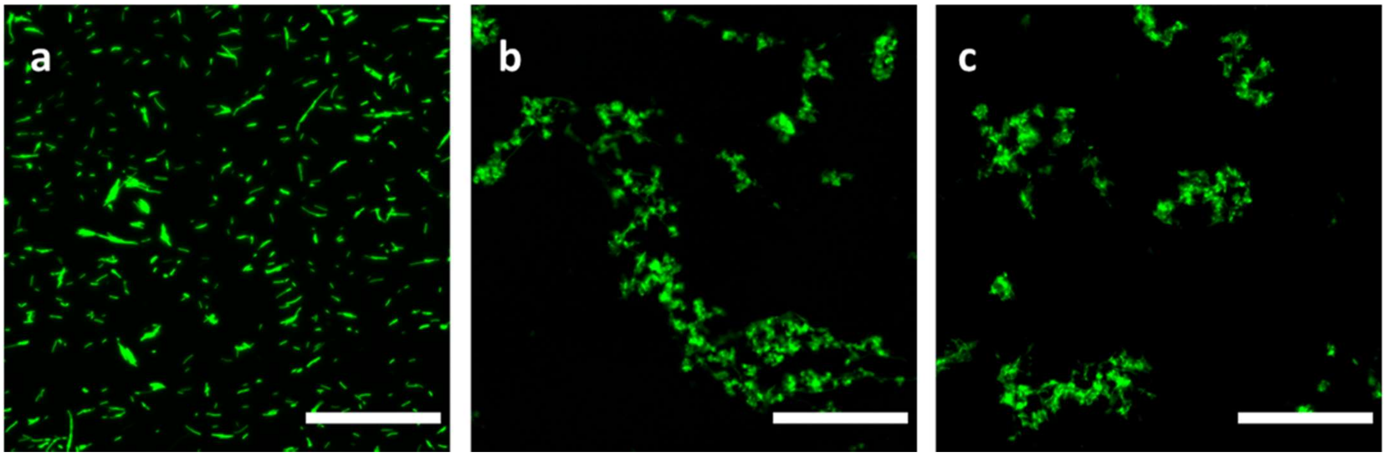

*Figure **8**: Widefield fluorescence imaging of induced sfGFP-expressing E. coli BL21(DE3) (green) grown overnight at 37 °C in suspension (LB/carbenicillin) with varying $CaCl_2$ supplementation and a 4-hour induction with L-arabinose before imaging. **a**) Negative control suspension without $CaCl_2$, showing the characteristic rod-like structure of E. coli dividing into short chains of cells. **b**) 50 mM $CaCl_2$ supplemented suspension showing clusters of deformed cells that are still capable of expressing sfGFP after overnight growth. **c**) 100 mM $CaCl_2$ supplemented suspension showing clusters of deformed cells that are again still capable of expressing sfGFP after overnight growth. Scale bars 100 μm.*

Confocal fluorescence – sfGFP mCherry dual ink lattices

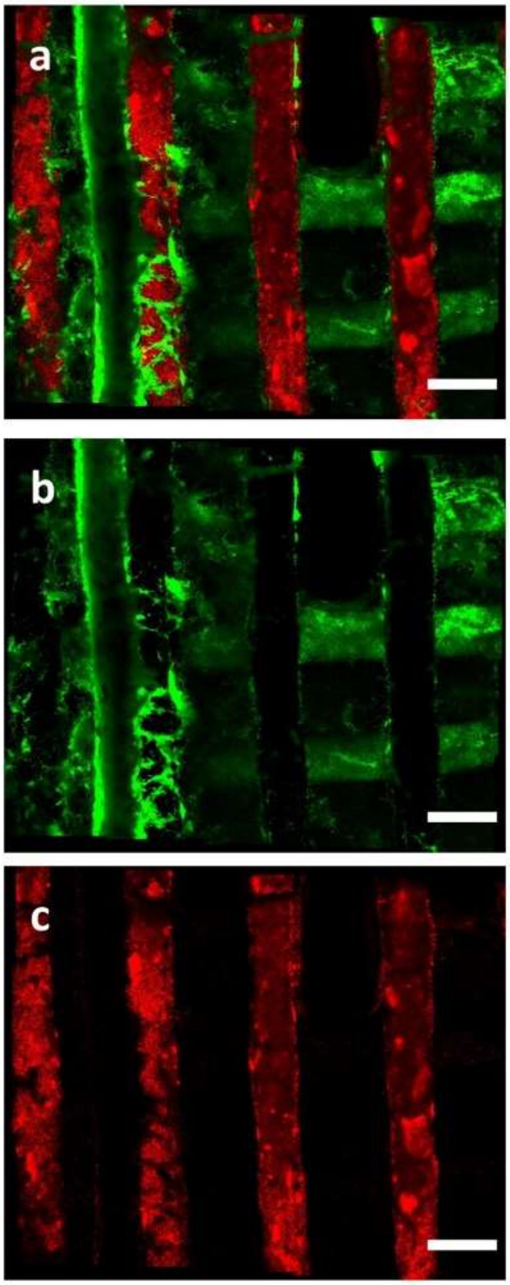

*Figure **9**: **a**) **b**) **c**): Sum of Z stack intensities from confocal fluorescence imaging of dual-extruded E. coli-sfGFP (green) and E. coli-mCherry (red) crosslinked lattices (bottom layer mCherry, orthogonal top layer sfGFP), induced with both L-arabinose (1w/v%) and IPTG (1 mM) during overnight shaking incubation at 37 °C in storage media **A**. Growth of each strain is constrained within the bulk of their starting filament. Significant biofilm formation on the surface has occurred, suggesting the cells are unable to move through the gel once embedded. **a**) Overlay of mCherry and sfGFP fluorescent signals. **b**) sfGFP signal. **c**) mCherry signal. Scale bars 1 mm. **d**) Combined sfGFP and mCherry fluorescence signal of horizontally adjacent fused filaments, showing minimal encroachment of each strain into the neighbouring filament suggesting immobilisation of bacteria within the bulk of the gel. Circular aperture used to reduce brightness. Scale bar 250 μm.*

CryoSEM

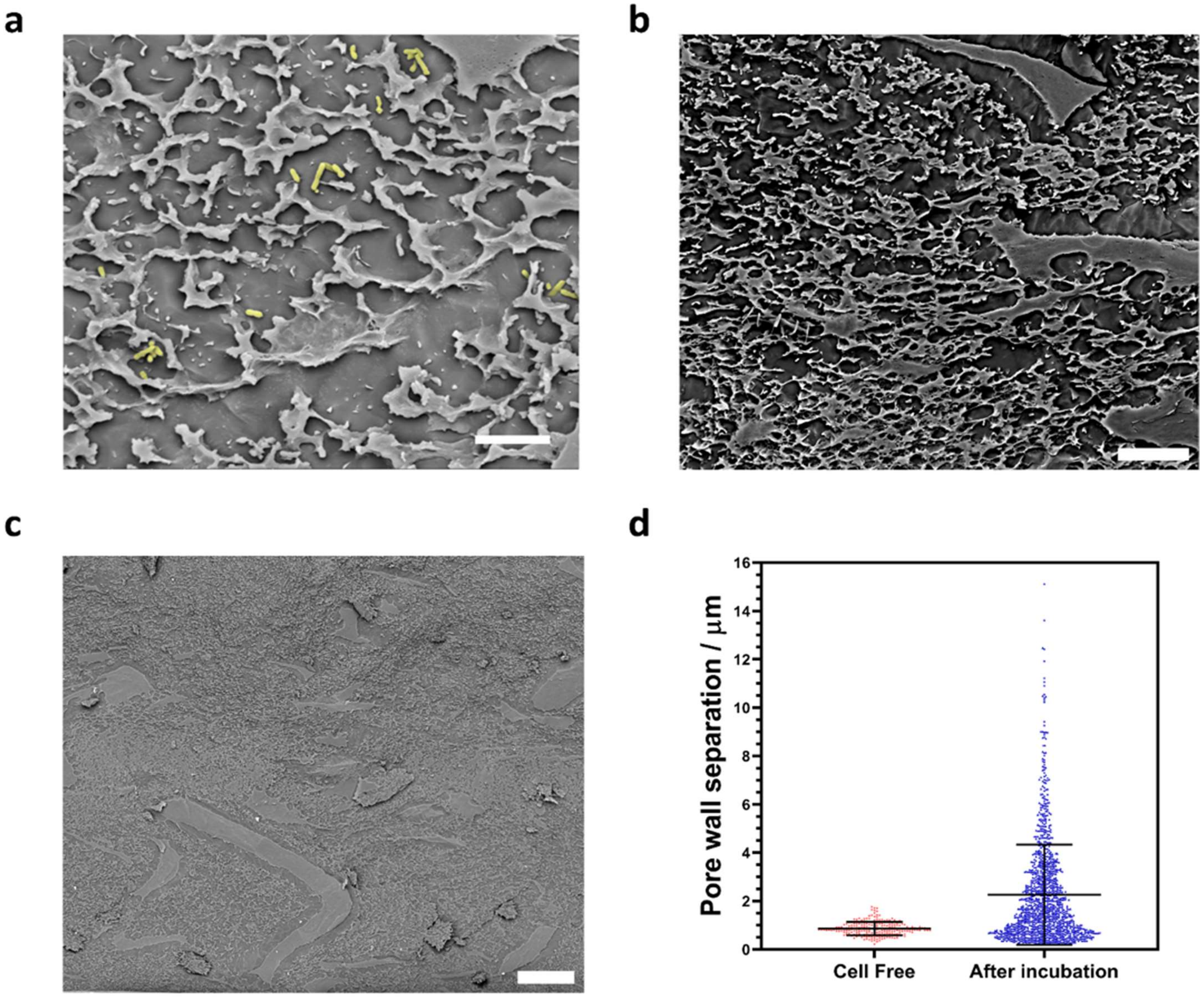

*Figure **10:** CryoSEM imaging (backscattered electrons) and pore size quantification. **a**) E. coli in porous structure. Scale bar 10 µm. **b**) Region of templated porous alginate. Scale bar 20 µm. **c**) Wide view, showing level of heterogeneity within the structure, with non-templated regions present. Scale bar 100 µm. E. coli false coloured yellow for clarity. **d**) Manual quantification of minimum pore wall separations averaged for cell-free and incubated cell-containing samples. Error bars are ±1 standard deviation, N = 240 and 1236 for 'cell free' and 'after incubation' respectively.*

OPC degradation

*ar*PTE structure

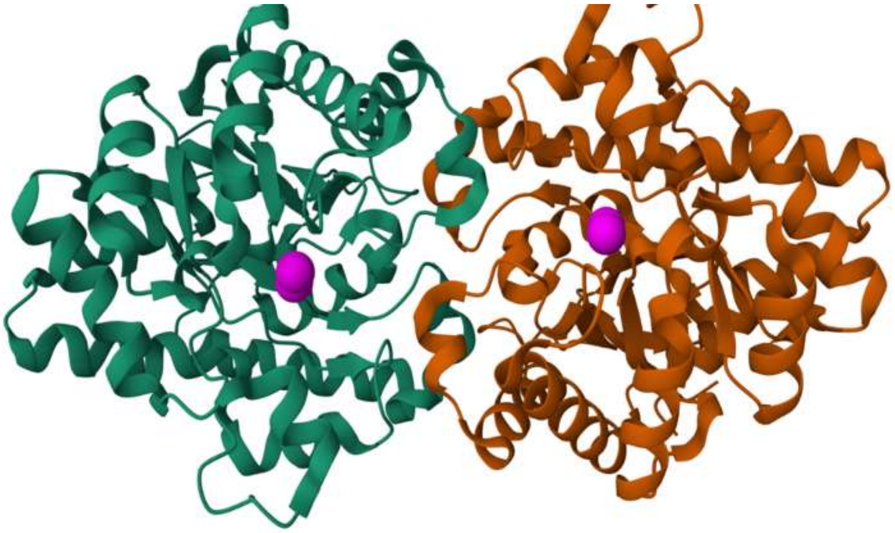

*Figure **11**: 3D structure of an arPTE dimer (green and orange monomers) joined through a hydrophobic binding domain. Pairs of divalent metal ions situated within the two active sites shown in purple. From PDB ID: 2D2J*

Mechanism of PTE catalysed Paraoxon hydrolysis

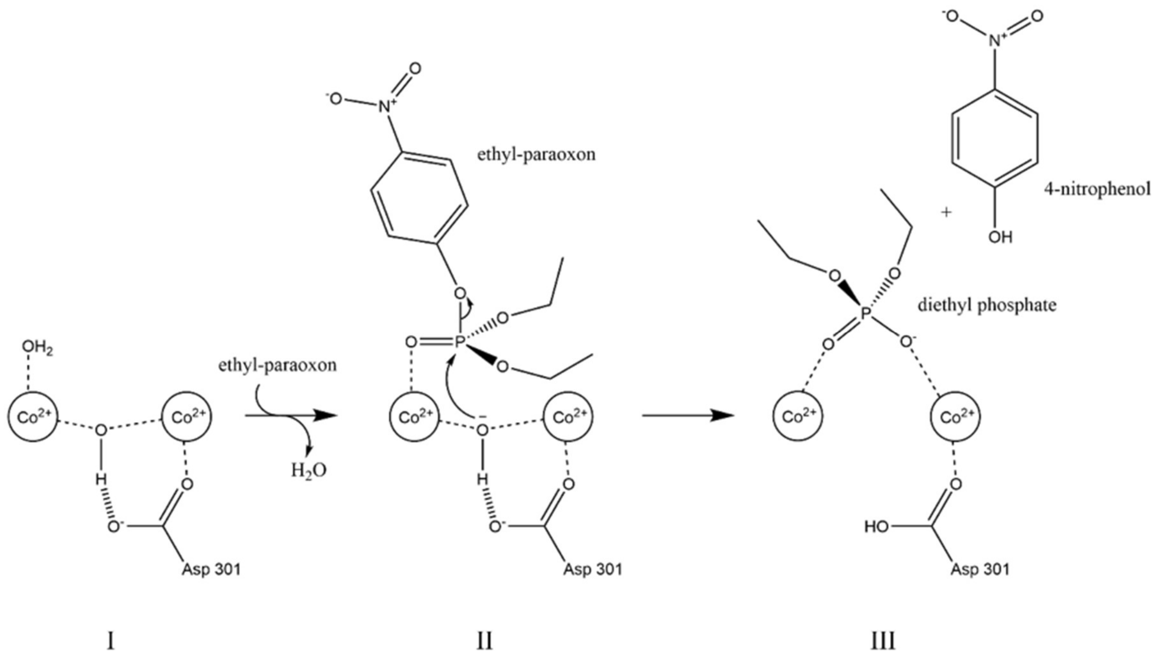

*Figure **12**: Reaction mechanism for the phosphotriesterase (PTE) catalysed hydrolysis of ethyl-paraoxon to 4-nitrophenol and diethyl phosphate, showing the empty, hydrated active site, with a pair of divalent metal ions (in this case Co^{2+} due to supplementation) bound in the active site by two histidine residues each (not shown in this mechanism for clarity) bridged by a hydroxyl nucleophile, with neighbouring asparagine base (**I**). The water molecule associated with the α-metal ion is displaced by the substrate (ethyl-paraoxon), with subsequent base catalysed nucleophilic attack by the bridging hydroxyl on the phosphorus centre of the substrate, cleaving the P-O ester bond (**II**), releasing the leaving group (4-nitrophenol) and diethyl phosphate acid. Adapted from the mechanism proposed by A. N. Bigley and F. M. Raushel. (Bigley, A. N. & Raushel, F. M. Biochim. Biophys. Acta - Proteins Proteomics **1834**, 443–453 (2013))*

Colorimetry data for quiescent 6 well ring reactiors with cell density variation and overnight growth with inducer or repressor.

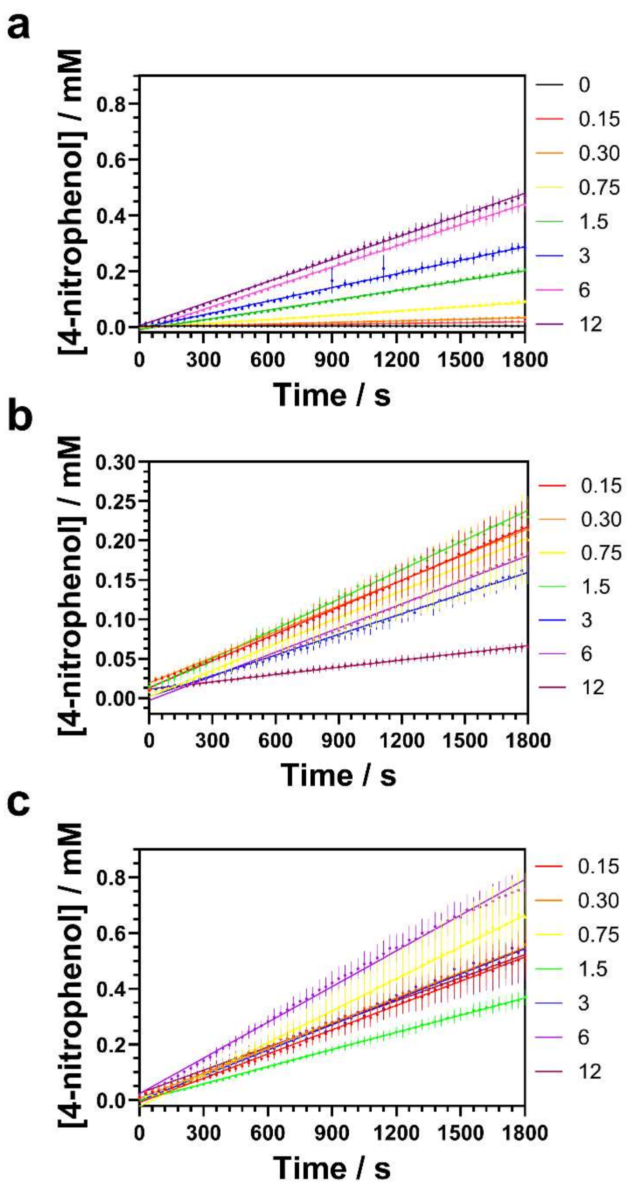

*Figure **13**: Colorimetric traces of 4-nitrophenolate production, measured as absorbance at 405 nm. Converted to concentration from absorbance using Beer Lambert Law, and the extinction coefficient of 4-nitrophenolate at pH 8 – 15550 $M^{-1}cm^{-1}$. **a**) 'Day 0' ring prints assessed immediately after crosslinking. **b**) 'Induced' ring prints, after overnight induction in Storage Media A with 1 mM IPTG supplement. **c**) 'Repressed' ring prints, after overnight induction in Storage Media A with 1% D-Glucose supplement.*

Colorimetry data for quiescent 6 well ring reactiors with varied SA/Vol, varied substrate hydrophobicity, and varied substrate concentration.

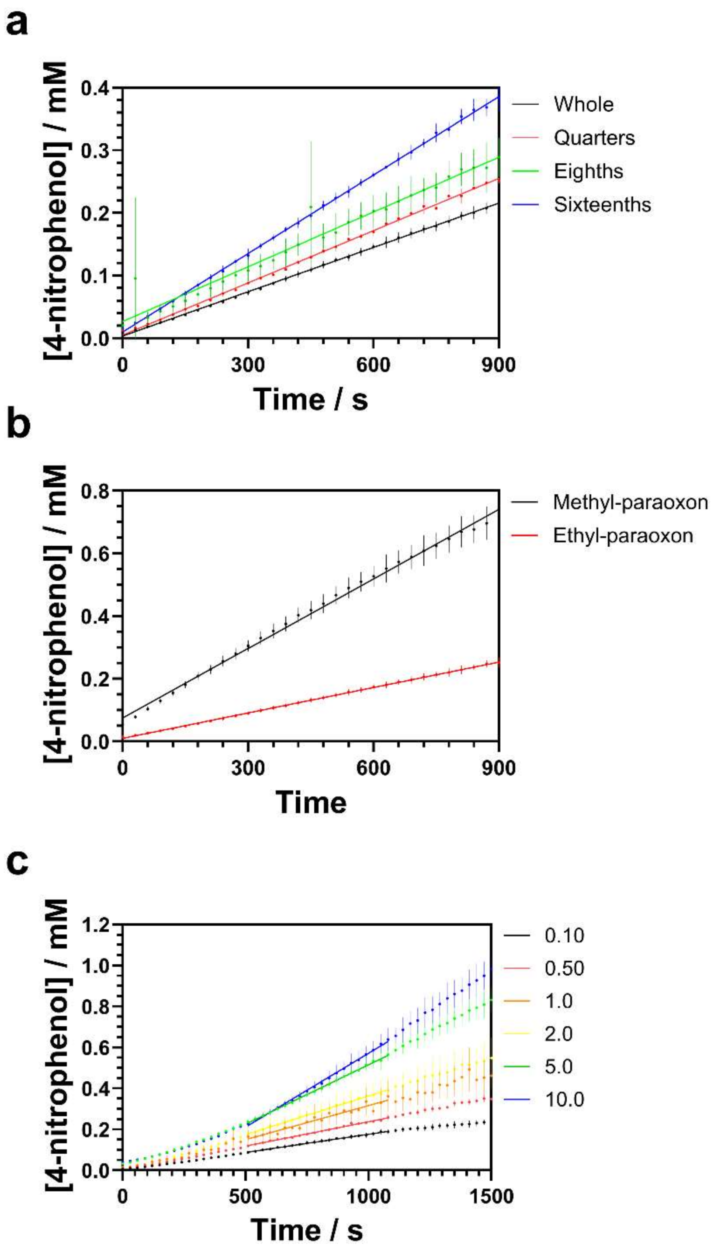

*Figure **14**: Colorimetric traces of 4-nitrophenolate production, measured as absorbance at 405 nm. Converted to concentration from absorbance using Beer Lambert Law, and the extinction coefficient of 4-nitrophenolate at pH 8 – 15550 $M^{-1}cm^{-1}$. **a**) OD 1.8 loaded 6 well ring prints with volume specific surface area varied by dividing the ring structures into equal partitions using a scalpel. **b**) OD 1.8 loaded 6 well ring prints challenged with ethyl- or methyl-paraoxon (1 mM). **c**) OD 1.8 loaded 6 well ring prints challenged with varying concentrations of ethyl-paraoxon*

Generation of 6 well ring structures for quiescent OPC hydrolysis.

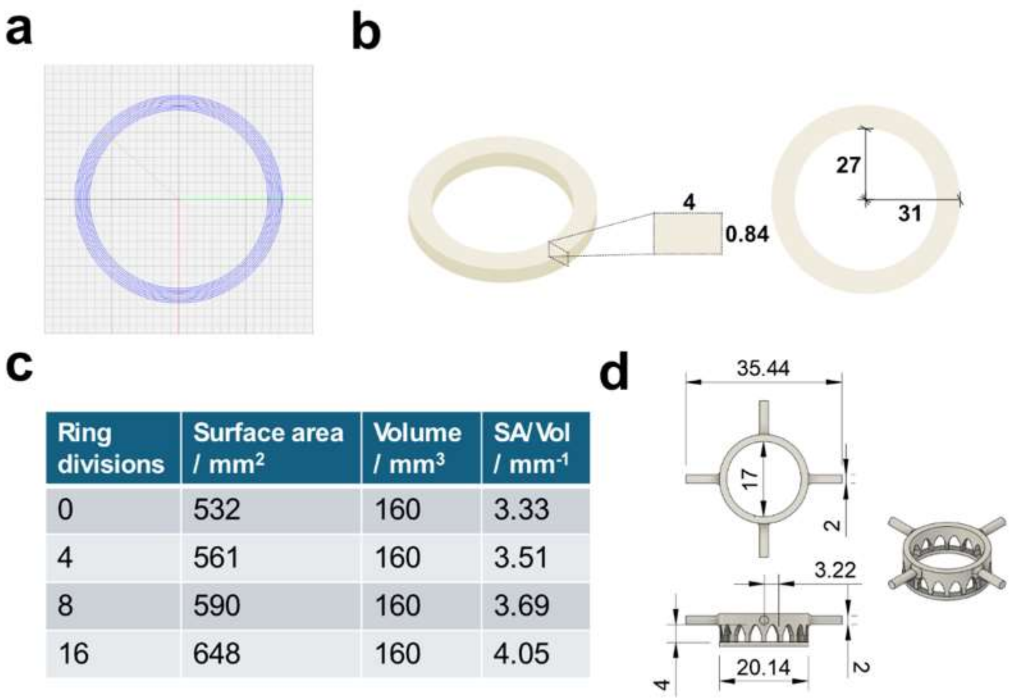

Ring divisions	Surface area / mm²	Volume / mm³	SA/Vol / mm⁻¹
0	532	160	3.33
4	561	160	3.51
8	590	160	3.69
16	648	160	4.05

*Figure **15**: Assessment of surface area:volume ratio impact on degradation rates. **a**) Gcode toolpath to generate six well rings using an 18 G needle with a layer height of 0.84 mm. Small grid squares are 1 mm x 1mm. **b**) the dimensions of the 3D printed rings. **c**) The volume specific surface areas (SA/Vol) generated by dividing the rings into equal sections using a scalpel, cutting through the cross section of the ring perpendicular to the top face. **d**) 3D render of resin printed insert used to prevent the ring from drifting across the beam path during colorimetric measurement, with dimensions in mm.*

OPC hydrolysis with varied cell density followed by induction or repression of *ar*PTE expression.

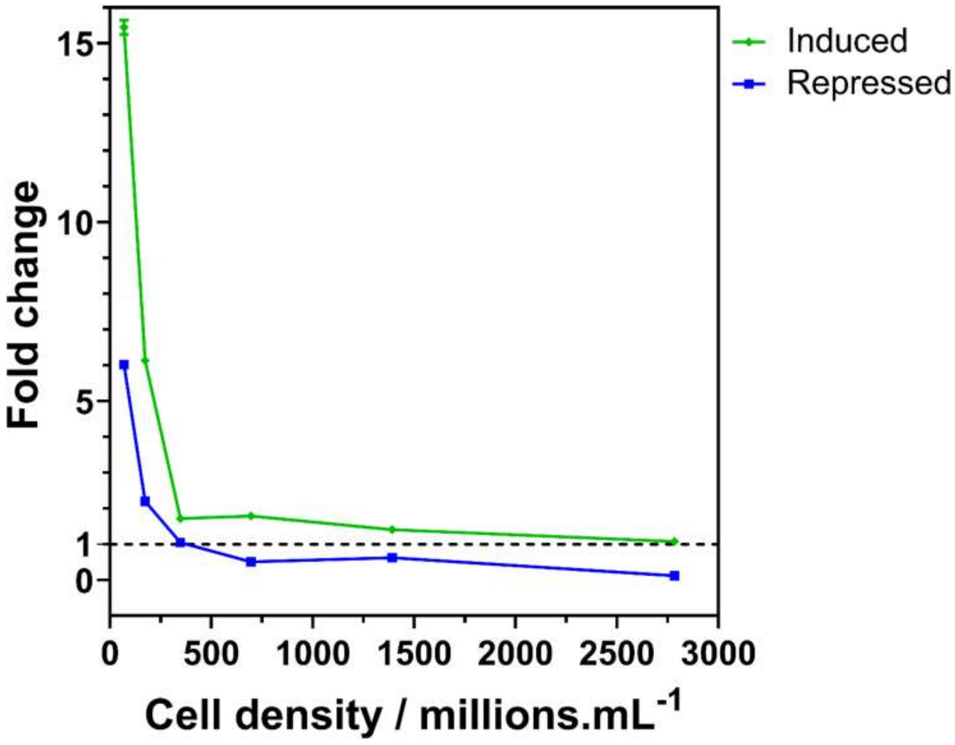

*Figure **16**: Fold change in ethyl-paraoxon hydrolysis rates of ring constructs with varied loading cell density that have been incubated overnight with inducer ('Induced', green, IPTG 1mM) or repressor ('Repressed', blue, D-glucose 1w/v%), relative to constructs of the same loading cell density measured immediately after cross-linking.*

MM kinetic characterisation of ELM and aqueous *ar*PTE

*Table **3**: Michaelis Menten kinetic values for non-crowded and BSA-crowded aqueous arPTE and six-well ring ELM activity with ethyl-paraoxon as substrate. Determined from colorimetric analysis of initial hydrolysis rates.*

	Non-crowded aqueous *ar*PTE	BSA-crowded aqueous *ar*PTE	Six-well, OD 1.8 loading, ELM
V_{lim} / μM·s^{-1}	0.55	0.66	0.38
K_m / mM	0.05	0.05	0.18
k_{cat} / s^{-1}	118.3	142.2	N/A

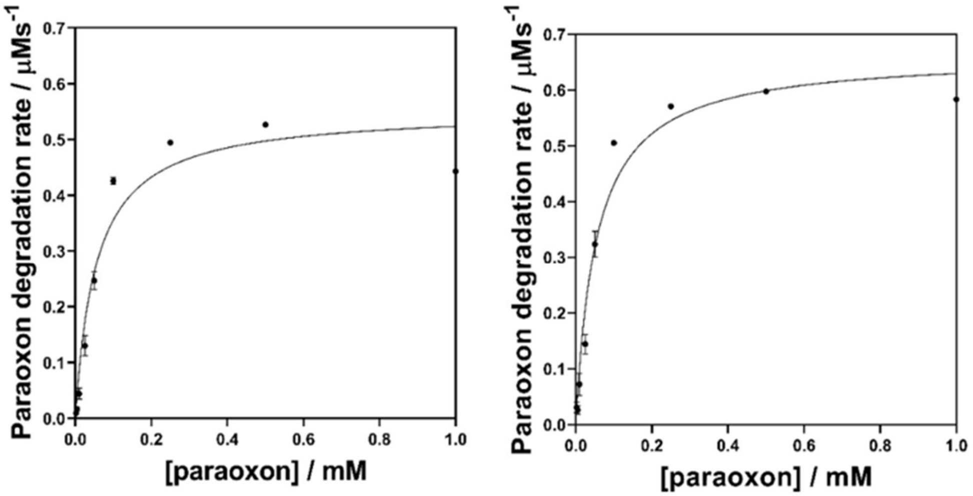

*Figure **17**: Ethyl-paraoxon degradation rate dependence on substrate concentration for **a**) non-crowded aqueous arPTE (4.67 nM), and **b**) Bovine Serum Albumin (BSA) crowded (10 mg·mL^{-1}) aqueous arPTE (4.67 nM), 4 replicates per concentration assessed. Error bars shown (if large enough to display) are standard errors of initial rates.*

Flow reactor casing

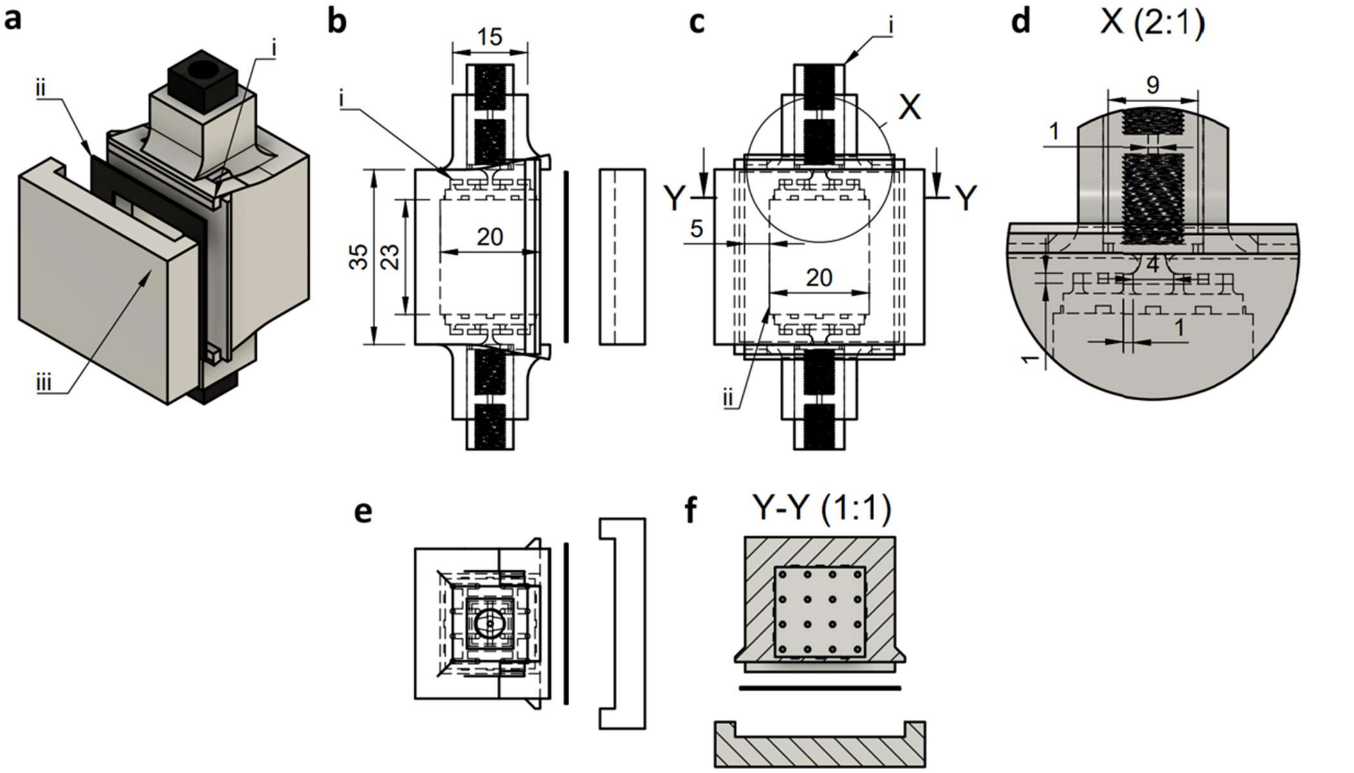

*Figure **18**: **a**) Shaded isometric view of the flow reactor design, **i**) The lip used to secure a panel closure, held in place with a clamp. **ii**) Neoprene gasket providing watertight seal around front panel. **iii**) Closure panel, also 3D printed from clear resin. **b**) Side wireframe projection of flow reactor, **i**) Fractal flow distributor, present as inlet and outlet. **c**) Front wireframe projection of reactor with detail view X, and section Y -Y marked; **i**) Threaded Delrin insert, glued in place to the top and bottom of the reactor using two-part epoxy resin. **ii**) Central cavity within the reactor to be loaded with 3D printed ELM; 20x20 mm square cross section, 23 mm long inlet-outlet. **d**) Detail X from **c** showing join between Delrin connector and fractal inlet; a 4 mm wide channel links into the 1 mm diameter fractal distributor, splitting one incoming flow into 16 equally space outlets on a square face. **e**) Top-down wireframe projection of reactor, showing main reactor body including fractal distributors, neoprene gasket, and printed closure panel. **f**) Section Y from **c** showing lower face of interior cavity, with outlet ports equally distributed across the square face. All dimensions in mm.*

Flow reactor practical setup

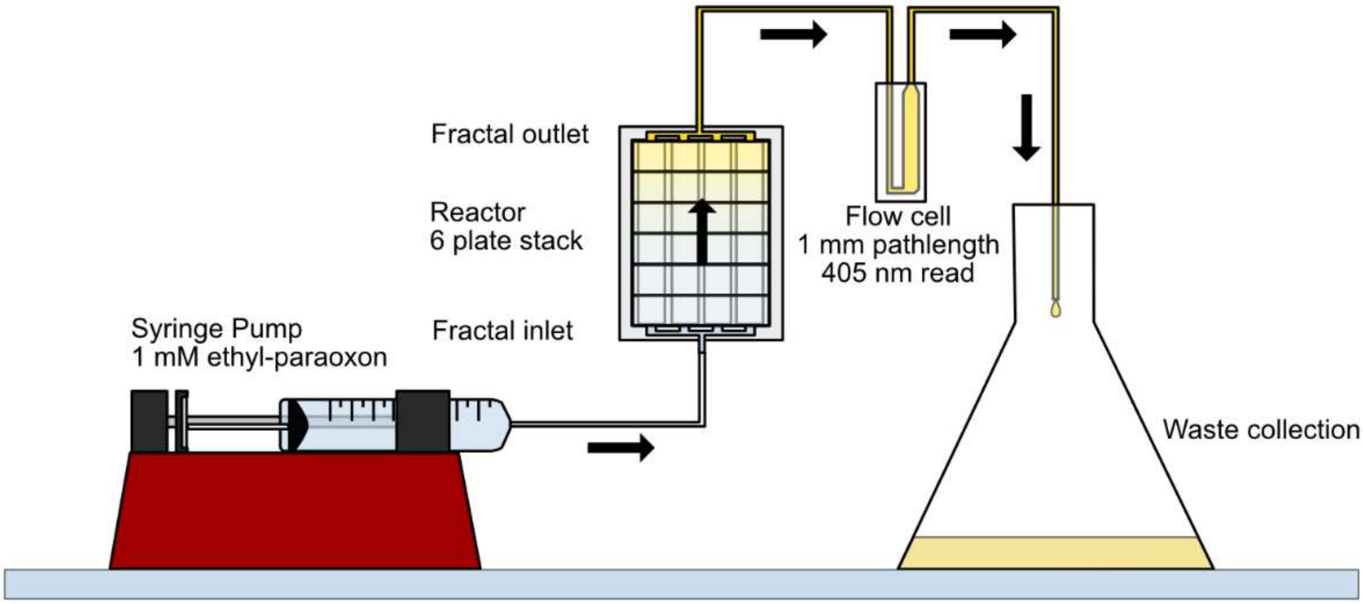

Figure ***19****: Experimental set-up used for flow reactor experiments. Fluid flow was generated using a screw-driven syringe pump, directed upwards through the 3D printed resin reactor to allow for removal of air during initial loading and equilibration. After interaction with the 3D printed ELM plates loaded in the reactor, solution passed through a 1 mm pathlength quartz flow cell allowing continuous measurement of the absorbance at 405 nm. After passing through the flow cell the semi-hydrolysed solution was collected and the remaining OPC destroyed using aqueous arPTE followed by bleach.*

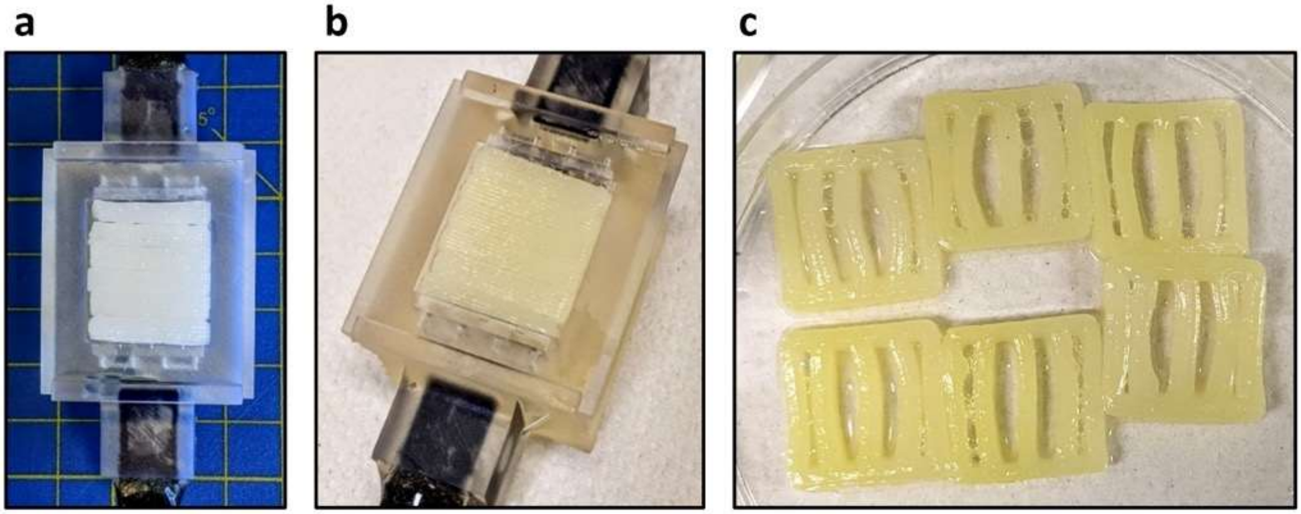

Figure ***20****: Catalytic microbial ELM reactor plates: Stacked within the reactor casing before (****a****) and after (****b****) use.* ***c****) After removal from the reactor case.*

One way ANOVA (Brown-Forsythe) for active 4-nitrophenol production at varied flow rates.

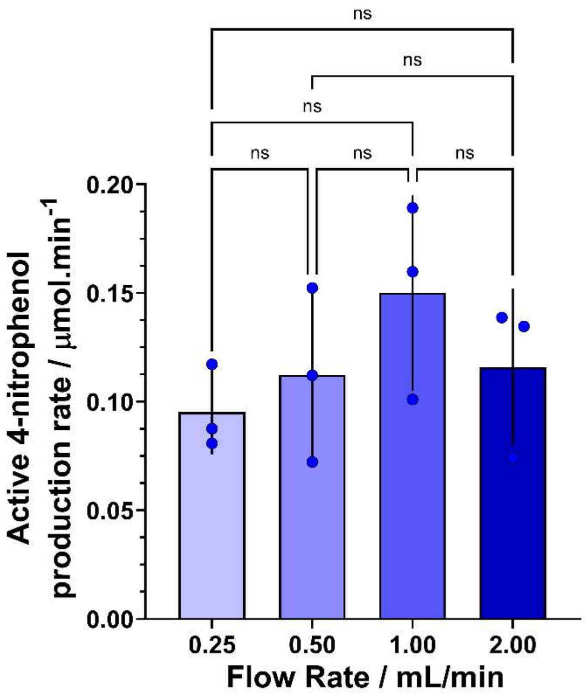

*Figure **21:** One-way ANOVA (Brown-Forsythe) results, with comparisons assessed between all pairs of flow rates, showing no significant difference in active 4-nitrophenol production rate across any pair of flow rates tested with the flow reactor (n=3, significance considered as P<0.05). Data from Figure 5d.*

sfGFP-*ar*PTE fusion creation and characterisation

*Table **4**: Fusion construct sequence and structure*

Construct	Structure	Sequence
sfGFP-<br>*ar*PTE	His6-tag<br>*Thrombin<br>cleavage site<br>[redacted] sfGFP<br>Linker arPTE<br>[redacted] [redacted]	M)GSSHHHHHHSSGLVPR*GSSHM[redacted]MSKGEELFTGVVPILVELDGDVNGHKFSVRGEGEGDATNGK<br>LTLKFICTTGKLPVPWPTLVTTLTYGVQCFSRYPDHMKRHDFFKSAMPEGYVQERTISFKDDGTYKTR<br>AEVKFEGDTLVNRIELKGIDFKEDGNILGHKLEYNFNSHNVYITADKQKNGIKANFKIRHNVEDGSVQLA<br>DHYQQNTPIGDGPVLLPDNHYLSTQSVLSKDPNEKRDHMVLLEFVTAAGITHGMDELYKGSEAAAKEA<br>AAKEAAAKGSMSMARPIGTGDLINTVRGPIPVSEAGFTLTHEHICGSSAGFLRAWPEFFGSRKALVEKA<br>VRGLRHARAAGVQTIVDVSTFDIGRDVRLLAEVSRAADVHIVAATGLWFDPPLSMRMRSVEELTQFFLR<br>EIQHGIEDTGIRAGIIKVATTGKATPFQELVLRAAARASLATGVPVTTHTSASQRDGEQQAIFESEGLSPS<br>RVCIGHSDDTDDLSYLTGLAARGYLVGLDRMPYSAIGLEGNASALALFGTRSWQTRALLIKALIDRGYKD<br>RILVSHDWLFGFSSYVTNIMDVMDRINPDGMAFVPLRVIPFLREKGVPPETLAGVTVANPARFLSPTVRA<br>S[redacted]

sfGFP-*ar*PTE Alphafold predicted structure

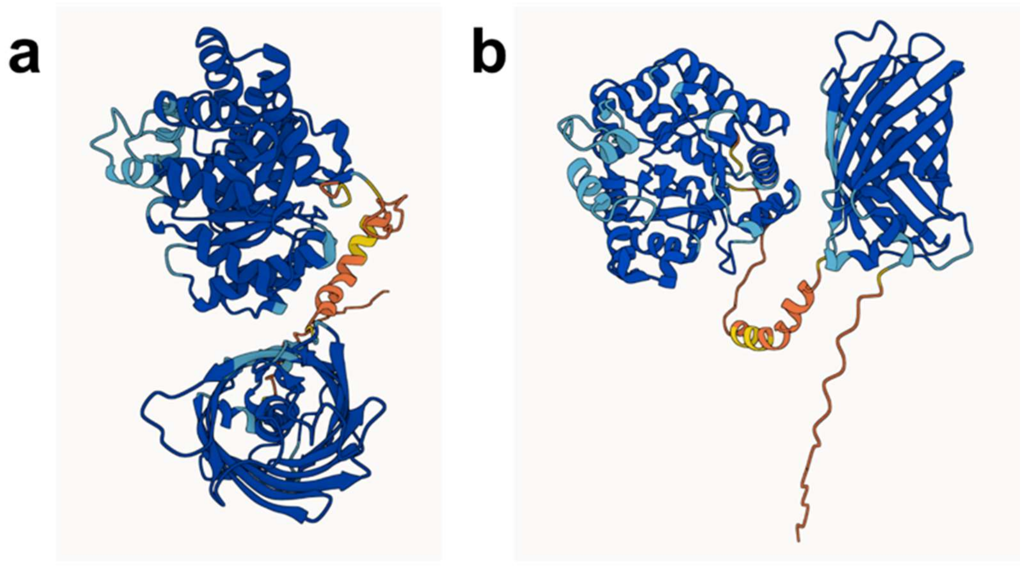

*Figure **22**: Alphafold 3D structure predictions for the created sfGFP-arPTE fusion protein based on intended sequence. **a**) Alphafold structure oriented along axis of the sfGFP barrel core. **b**) Alphafold structure oriented along axis running into the substrate binding pocket of arPTE. The linker region can be seen between the two proteins and the long His6 purification tag chain below the structure. Coloured according to the per residue model confidence (as predicted local-distance difference test or pLDDT score) from 0 – 100: Dark blue 90-100 (very high); light blue 70-90 (confident); yellow 50-70 (low); orange 0-50 (very low).*

sfGFP-*ar*PTE circular dichroism

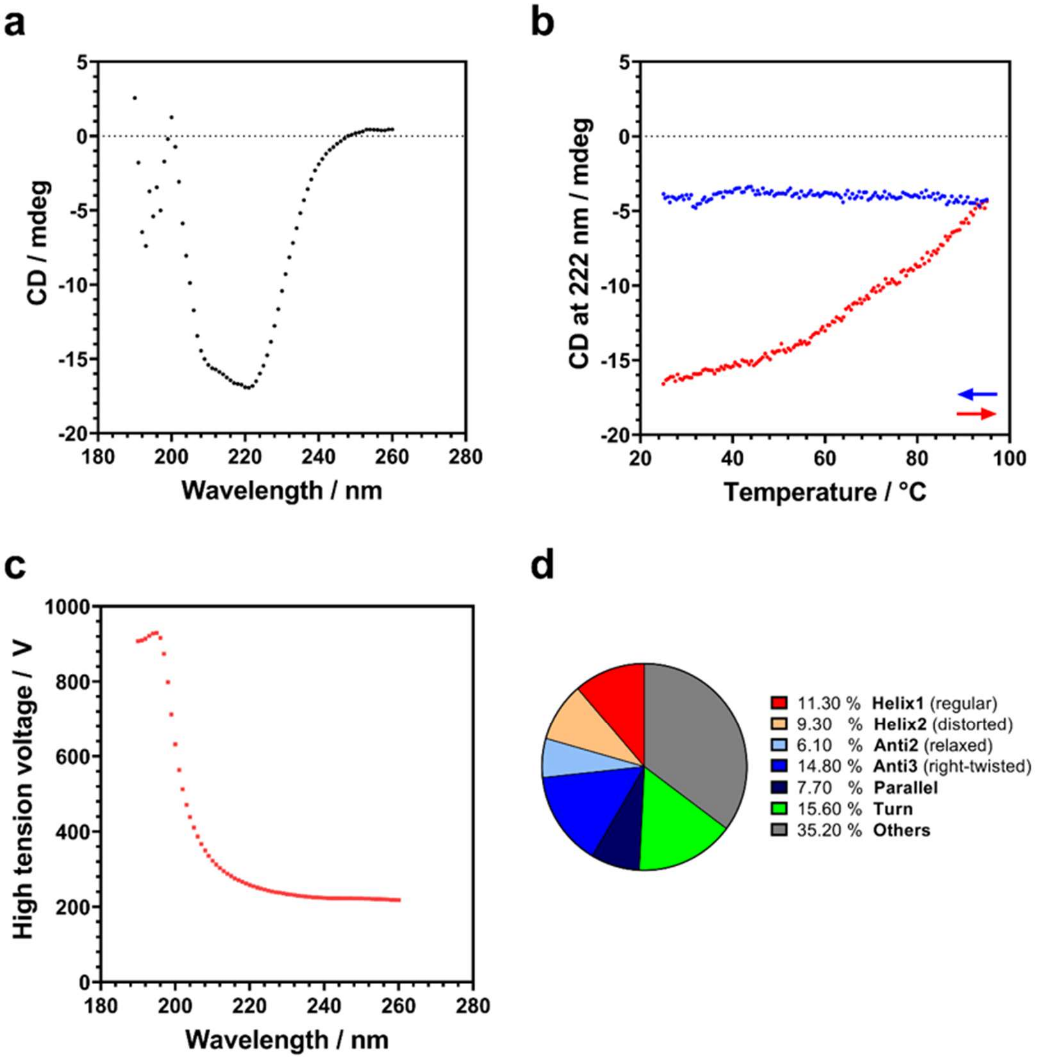

*Figure **23**: sfGFP-arPTE structure characterisation by circular dichroism. **a**) Circular dichroism of purified sfGFP-arPTE (SEC peak 1 at ~200 mL). **b**) Circular dichroism at 222 nm during temperature ramp up (red) and down (blue). **c**) High tension voltage applied to the detector to account for received signal intensity variation as wavelength was adjusted in **a**. **d**) Estimated secondary structure proportions resulting from BeStSEL model.*

sfGFP-*ar*PTE functional imaging

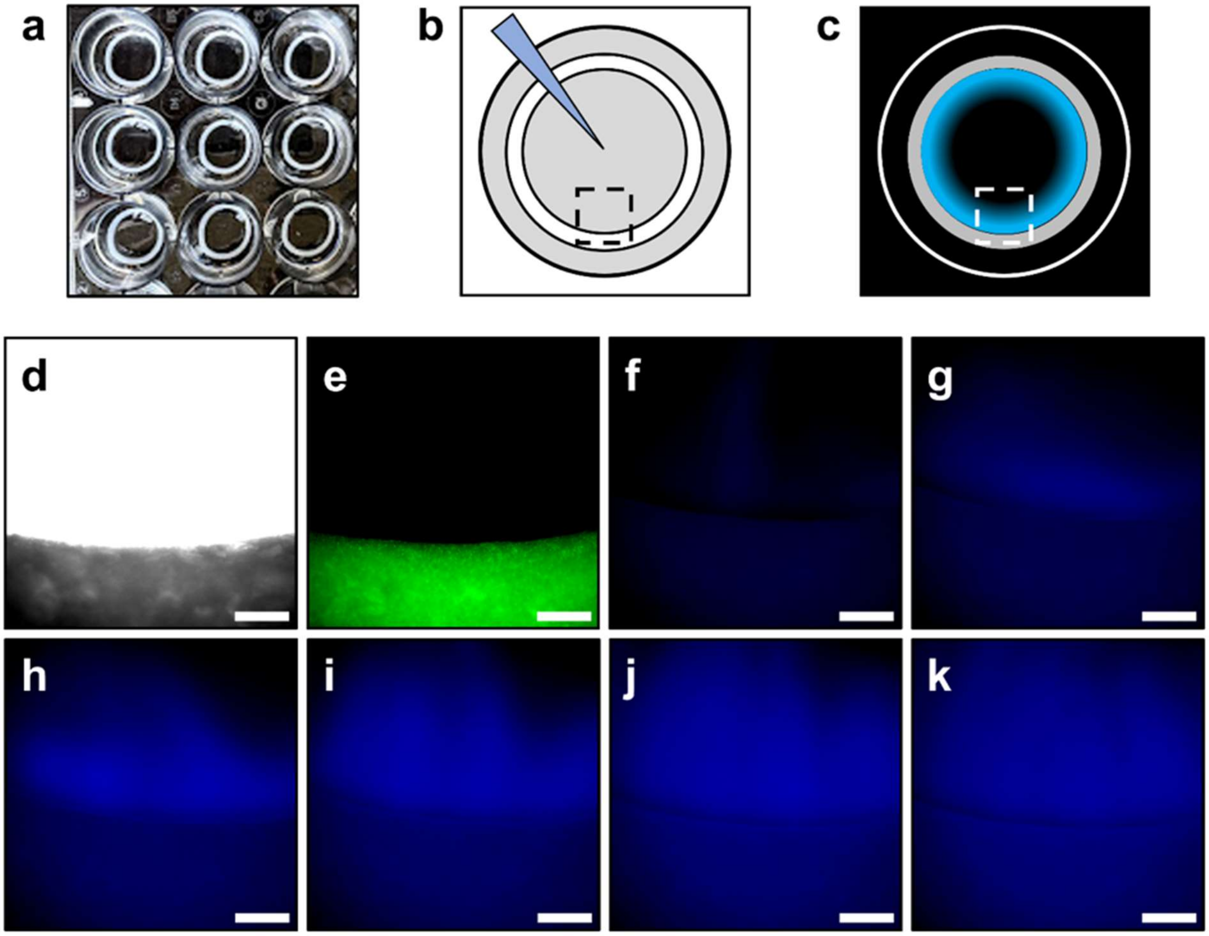

*Figure **24**: Preliminary localisation of sfGFP-arPTE retention, and time-course imaging of chlorferon production and diffusion. **a**) 24-well ring prints after crosslinking, containing sfGFP-arPTE expressing E. coli. **b**) Schematic showing location of Coumaphos addition in the centre of the well (blue 'pipette tip') and top-down widefield imaging field of view (black dashed rectangle). **c**) Schematic representation of the expected evolution of chlorferon production and diffusion away from the ELM interior surface, with imaging field of view (white dashed rectangle). **d**) Brightfield image within the field of view identified in **b** and **c**. **e**) Green fluorescent signal recorded at the ring edge, focused on the middle of the ELM ring. **f**-**k**) Blue fluorescence recorded at the ring interior, with one second imaging frequency; shown here are representative one-minute intervals. Scale bars 500 µm.*

Mechanism of PTE catalysed Coumaphos hydrolysis

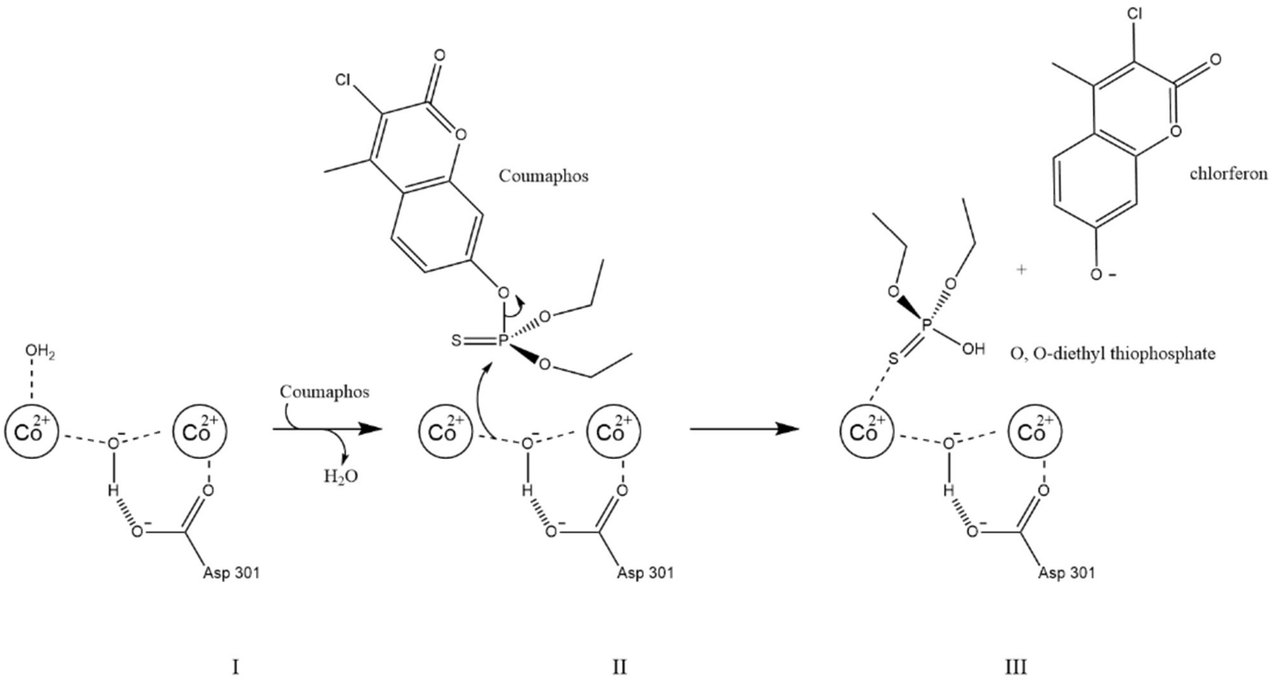

*Figure **25**: Reaction mechanism for the phosphotriesterase (PTE) catalysed hydrolysis of Coumaphos to fluorescent chlorferon and O, O-diethyl thiophosphate.*

Reaction-diffusion imaging experimental sequence

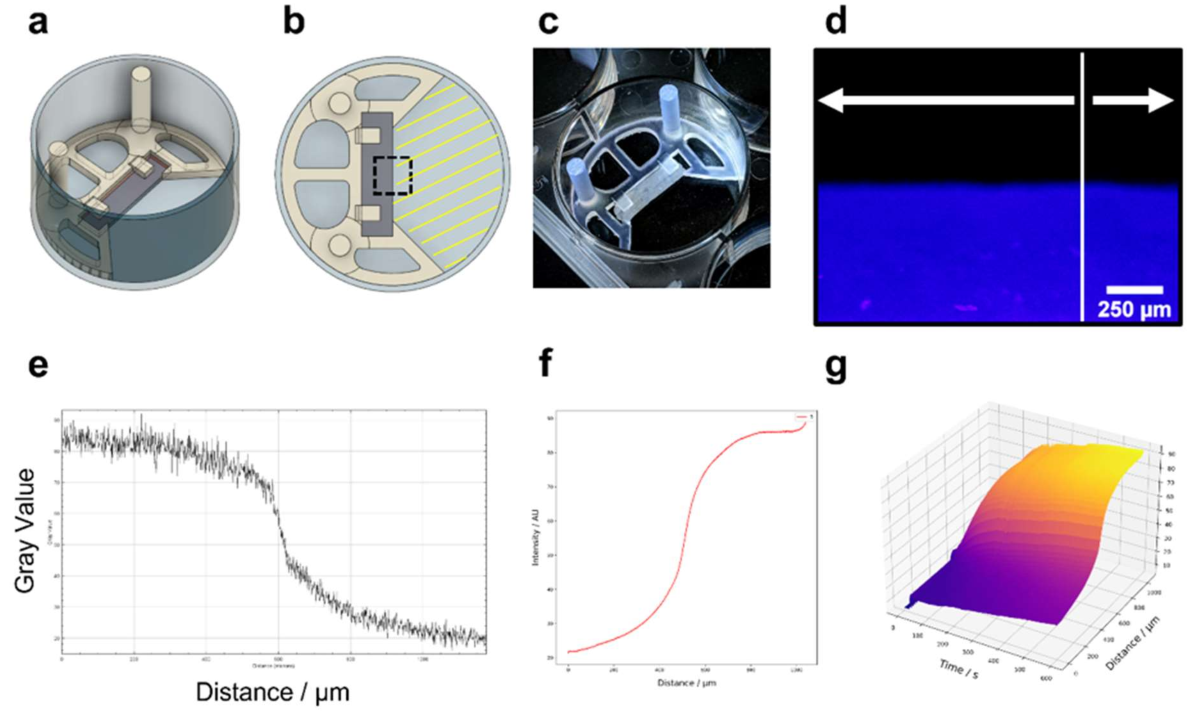

*Figure **26**: Experimental setup and data processing sequence for imaging of the production and diffusion of chlorferon within the ELM. **a**) Isometric view of initial gel trap design (white) inside a six-well plate. **b**) Top-down view of the design, showing intended substrate solution reservoir (yellow hatching) and field of view for time-course imaging (black dashed square). **c**) 3D printed trap with sheet of printed ELM hydrogel underneath. **d**) Representative preliminary image of the ELM-solution interface at an early timepoint after substrate addition. The white arrows demonstrate the axis along which intensity profiles (white line) that run away from the interface were averaged. **e**) Representative single intensity profile with interface roughly halfway through the image field of view. **f**) Averaged intensity profile for a single timepoint. **g**) Average intensity profiles for an eleven-minute time course collated into a 3D kymograph (Fluorescence intensity as both z axis height and assigned to purple-orange-yellow colour map). For final data processing, the intensity profile immediately before substrate addition was subtracted as a baseline value.*
